# Integrative metagenomic and metabolomic analysis reveals a gut microbiota-metabolite-immune axis in pediatric allergic rhinitis with functional constipation

**DOI:** 10.3389/fcimb.2026.1779298

**Published:** 2026-05-26

**Authors:** Hui Wu, Lindong Shi, Chunyan Wang, Yushan Liang, Congfu Huang

**Affiliations:** 1 Department of Child Health and Rehabilitation, Maternal and Child Health Hospital of PanYu District, Guangzhou, China; 2 Department of Pediatrics, Affiliated Longgang Central Hospital of Shantou University Medical College, Shenzhen, China; 3 Department of Pediatrics, Shenzhen Fourth People’s Hospital, Shenzhen Sami Medical Center, Shenzhen, China; 4 Department of Pediatrics, Longgang Maternity and Child Institute of Shantou University Medical College, Longgang District Maternity & Child Healthcare Hospital of Shenzhen City, Shenzhen, China

**Keywords:** allergic rhinitis, amino acids, functional constipation, gut microbiota, metagenomics, microbiota-metabolite axis, targeted metabolomics

## Abstract

**Objective:**

This study aimed to delineate the alterations in the gut microbiome and host amino acid metabolism in children with comorbid allergic rhinitis and functional constipation (ARFC), and to explore their links with clinical allergy markers.

**Methods:**

We performed shotgun metagenomic sequencing and amino acid-targeted metabolomics on fecal samples from 19 children with ARFC and 16 age-matched healthy controls (HC). Microbial community structure, differentially abundant taxa, and metabolic profiles were analyzed. Integrative analyzes, including correlation networks and machine learning modeling, were employed to investigate microbiota-metabolite-host interactions.

**Results:**

Significant beta-diversity distinction was found between ARFC and HC gut microbiota (PCoA R^2^=0.228, P = 0.001). ARFC children exhibited enrichment of mucin-degrading Bacteroidota (e.g., Bacteroides, Phocaeicola) and depletion of beneficial Bacillota (e.g., Bifidobacterium, Blautia). Metabolomics identified 50 differentially abundant metabolites, with widespread downregulation of immunomodulatory amino acids including L-glutamine and γ-aminobutyric acid (GABA). Enriched pathways involved mTOR and FoxO signaling, and neurotransmitter synapses. Integration revealed significant correlations between specific microbial genera (e.g., Bacteroides, Proteus) and metabolites (e.g., kynurenine), and between gut species (e.g., Bacteroides thetaiotaomicron) and serum IgE levels. A machine learning model integrating key microbial and metabolic features, evaluated under a rigorous leave-one-out cross-validation framework, demonstrated robust discriminative performance in this cohort (AUC = 0.946).

**Conclusion:**

This multi-omics study unveils a distinct “gut dysbiosis-metabolite dysregulation-immune dysfunction” axis in ARFC children. The synergistic shift towards a mucolytic, pro-inflammatory microbiota alongside deficient immunomodulatory metabolite production, which correlates with clinical allergy markers, provides a novel mechanistic framework for this comorbidity and highlights potential diagnostic biomarkers for future validation.

## 1 Introduction

Allergic rhinitis (AR) ranks among the most prevalent chronic inflammatory diseases in the pediatric population, with a persistently rising global prevalence that imposes a significant public health burden (Wise et al., 2018). Its pathogenesis involves genetic susceptibility, environmental exposures (e.g., allergens, air pollutants), and complex immune dysregulation, ultimately leading to a predominant T helper 2 (Th2) immune response and IgE-mediated inflammation (Bousquet et al., 2025).

The “gut–lung axis” has emerged as a pivotal framework for elucidating the pathogenesis of respiratory allergic diseases (Renz and Skevaki, 2021). As the body’s largest microbial ecosystem, the gut microbiota critically orchestrates immune development and maintains mucosal barrier integrity (Renz and Skevaki, 2021). Accumulating evidence underscores that early-life gut microbiota dysbiosis is a key risk factor for the development of childhood allergic disorders, including asthma, atopic dermatitis, and AR (Fujimura et al., 2016; Stokholm et al., 2018). Proposed mechanisms encompass impaired regulatory T (Treg) cell differentiation, deficient production of immunomodulatory metabolites such as short-chain fatty acids (SCFAs), and increased intestinal permeability, collectively fostering a state of immune dysregulation and systemic inflammation (Tan et al., 2014).

Clinically, children with AR frequently experience comorbid functional gastrointestinal disorders, with functional constipation (FC) being notably prevalent. Epidemiological data indicate that approximately 20–30% of children with AR also meet the diagnostic criteria for FC (Wang et al., 2025). This AR and FC comorbidity (hereafter ARFC) not only aggravates nasal symptoms and diminishes quality of life but also poses challenges for clinical management (de Bruijn et al., 2022). While gut microbiota dysbiosis has been independently linked to both AR and FC (Wan et al., 2023; Feng et al., 2024), it remains unknown whether ARFC constitutes a distinct clinical entity driven by shared microbial and metabolic disturbances. Elucidating this potential synergistic axis is crucial for advancing both pathophysiological understanding and therapeutic strategies.

Technological advances now enable deep mechanistic insights. Shotgun metagenomic sequencing surpasses the resolution limits of 16S rRNA profiling, allowing comprehensive characterization of the gut microbiome’s taxonomic and functional landscape (Quince et al., 2017). Complementarily, targeted metabolomics provides precise quantification of specific metabolite classes, such as amino acids, offering a direct readout of microbial community function and host metabolic status (Pang et al., 2022). The integration of these two approaches—constructing “microbiota–metabolite–host” interaction networks—has proven powerful for unraveling the etiology of complex multifactorial diseases (Zierer et al., 2018).

Despite this conceptual and methodological progress, a systematic, multi-omics characterization of the gut ecosystem in children with ARFC is currently absent. Notably, recent work has further delineated how gut microbiota-derived metabolites, including amino acids and their derivatives, can regulate local and systemic immune responses (Dang and Marsland, 2019; Durack and Lynch, 2019). This reinforces the imperative to define specific microbial and metabolic drivers in allergic–gastrointestinal comorbidities. To address this gap, we conducted the first integrated metagenomic and amino acid-targeted metabolomic study in pediatric ARFC. Our specific objectives were to: 1) compare gut microbiota structure and function between ARFC children and healthy controls (HC); 2) characterize perturbations in fecal amino acid metabolism and identify associated biological pathways; and 3) construct integrative correlation networks linking specific gut microbes, differential metabolites, and clinical allergy markers. This work seeks to establish a novel “gut microbiota–metabolism–immunity” axis underlying ARFC and to identify candidate targets for future diagnostic and therapeutic development.

## 2 Materials and methods

### 2.1 Study participants and study design

We conducted a case-control study at the pediatric outpatient clinic of Longgang District Maternity & Child Healthcare Hospital, Shenzhen, China. Between January and December 2024, children diagnosed with AR (Cheng, 2022) were screened for eligibility. Those who also met the diagnostic criteria for FC (Hyams et al., 2016) were enrolled into the ARFC group. Age-, sex-, and habitual diet-matched healthy children, with no history of allergic or chronic gastrointestinal diseases, were recruited from the local community to serve as healthy controls (HC). Habitual dietary intake was assessed using a validated semi-quantitative food frequency questionnaire (FFQ) adapted for Chinese preschool children, which queried consumption frequency and portion sizes of 83 food items over the preceding three months. The FFQ was administered to parents/guardians by trained research staff during the enrollment visit. To enhance accuracy and capture recent intake patterns, a single 24-hour dietary recall was also completed at the time of fecal sample collection. Nutrient intakes were calculated using the Chinese Food Composition Database. Dietary fiber, total energy, and total protein intake—as well as the ratio of animal-derived to plant-derived protein—were extracted as key matching and descriptive variables. All participants were confirmed to have no history of antibiotic, probiotic, or prebiotic use within three months prior to enrollment. Clinical characterization of ARFC participants included assessment of allergic rhinitis severity using the Total Nasal Symptom Score (TNSS), which evaluates nasal congestion, sneezing, rhinorrhea, and nasal itching on a 0–3 scale (range: 0–12). Disease control status was classified according to Allergic Rhinitis and its Impact on Asthma (ARIA) guidelines as intermittent or persistent. Functional constipation severity was evaluated using the Bristol Stool Form Scale (BSFS) and defecation frequency based on Rome IV criteria. Comprehensive clinical parameters for both groups are summarized in **Supplementary Table 3**. The study protocol was approved by the Ethics Committee of Longgang District Maternity & Child Healthcare Hospital (Approval No: KYXMLL-01-CZGC-14-2-1). Written informed consent was obtained from the legal guardians of all participants prior to enrollment. The study was registered with the Chinese Clinical Trial Registry (Registration No: ChiCTR2400085982).

### 2.2 Sample size calculation and participant characteristics

The sample size was estimated *a priori* using G*Power 3.1 software. Preliminary data from a pilot study (n=6 per group) indicated a large effect size (Cohen’s d = 1.2) for the primary outcome of gut microbiota β-diversity (assessed by PERMANOVA on Bray-Curtis dissimilarity). To achieve 80% power with a two-sided α of 0.05, a minimum of 12 participants per group was required. Anticipating potential technical failures or data exclusions, we enrolled 19 children with ARFC and 16 HC children, ensuring the final analyzable sample size exceeded the calculated minimum. All participants had no history of antibiotic, probiotic, or prebiotic use within three months prior to enrollment. Individuals with immunodeficiency, organic gastrointestinal diseases, or other chronic systemic diseases were excluded. The baseline demographic and clinical characteristics of the two groups are summarized in **Table 1**.

**Table 1:** Baseline characteristics of the ARFC group and HC group.

Characteristic	HC group (n=16)	ARFC group (n=19)	*P*-value
Age (years), mean ± SD	4.6 ± 0.6	4.9 ± 0.7	0.53
Gender (male/female)	9/7	13/6	0.36
Total energy intake (kcal/day)	1356 ± 184	1412 ± 203	0.47
Total protein intake (g/day)	48.3 ± 8.2	51.7 ± 9.5	0.31
Animal/plant protein ratio	1.42 ± 0.37	1.51 ± 0.42	0.52
Dietary fiber (g/day), mean ± SD	12.5 ± 1.6	13.2 ± 1.4	0.74
Antibiotic use (past 3 months), n (%)	0 (0%)	0 (0%)	–

### 2.3 Fecal sample collection and processing

#### 2.3.1 Sample collection

Following a 12−hour fast, fecal samples were collected using a standardized rectal swab protocol with sterile flocked swabs. This approach was selected to enable immediate sample stabilization and rapid cryopreservation—critical for metabolomic analysis—while circumventing the logistical challenges of spontaneous stool collection in young children. Briefly, after perianal cleansing, the swab was inserted 4–5 cm into the rectum, rotated gently to obtain mucosal−luminal interface material, and immediately transferred to a sterile cryovial. All samples were flash−frozen in liquid nitrogen and stored at −80 °C within 30 minutes of collection until nucleic acid extraction and metabolomic analysis.

#### 2.3.2 Contamination controls

To monitor potential contamination introduced during sampling, a collection blank (a sterile flocked swab exposed to ambient air for an equivalent duration) was processed in parallel with each batch of clinical samples. Both fecal swabs and collection blanks underwent identical downstream processing, including DNA extraction, library preparation, and sequencing. Taxa disproportionately enriched in collection blanks relative to biological samples were flagged as potential contaminants and excluded from downstream analyzes.

### 2.4 Metagenomic sequencing and bioinformatic analysis

#### 2.4.1 DNA extraction and library preparation

Total genomic DNA was extracted from approximately 200 mg of each fecal sample using the FastPure Stool DNA Isolation Kit (Magnetic bead) (Meiji Biomedical, China) following the manufacturer’s instructions. DNA concentration and purity were assessed using a NanoDrop 2000 spectrophotometer (Thermo Fisher Scientific, USA), and integrity was checked by 1% agarose gel electrophoresis. Qualified DNA was randomly sheared to an average fragment size of ~400 bp using a Covaris M220 focused-ultrasonicator (Covaris, USA). Sequencing libraries were constructed using the NEXTFLEX Rapid DNA-Seq Kit (Bioo Scientific, USA). Library quality was evaluated using an Agilent 2100 Bioanalyzer (Agilent Technologies, USA).

#### 2.4.2 Contamination controls and quality assurance

To identify and mitigate potential reagent- and laboratory-derived contaminants, a DNA extraction blank (nuclease-free water processed in parallel with fecal samples) and a no-template library preparation control were included with each analytical batch. Following sequencing, the proportional representation of all detected taxa in blank controls was compared against that in biological samples. Taxa whose mean relative abundance in blank controls exceeded 25% of their abundance in the lowest-quartile biological samples were flagged as probable contaminants and subsequently removed from the feature table prior to statistical analysis. Furthermore, the prevalence and relative abundance of all reported differentially abundant taxa were manually verified against the blank control profiles to ensure that no biological inferences were confounded by contaminating sequences.

#### 2.4.3 High-throughput sequencing and data processing

Paired-end sequencing (150 bp) was performed on an Illumina NovaSeq X Plus platform (Illumina, USA) by Majorbio Bio-Pharm Technology Co., Ltd. (Shanghai, China). To ensure adequate coverage for robust taxonomic profiling, sequencing was targeted to achieve a minimum depth of 20 million high-quality read pairs per sample. Raw sequencing reads were subjected to quality control using fastp (v0.20.0) to remove adapter sequences and low-quality reads (length < 50 bp or average Phred quality score < 20) (Enaud et al., 2020). The mean number of high-quality, non-host reads retained per sample post-filtering was 21.5 ± 3.2 million (mean ± SD), which satisfies the recommended minimum depth for comprehensive characterization of fecal microbial communities. Host-derived reads were identified and removed by aligning the clean reads to the human reference genome (hg38) using BWA (v0.7.17) (Chunxi et al., 2020). *De novo* metagenomic assembly of the remaining high-quality reads was performed using MEGAHIT (v1.2.9) (Li et al., 2015) with default parameters. Assembly quality was assessed using QUAST (v5.0.2), yielding the following summary metrics across all samples: total assembled length of 2.1 ± 0.8 Gbp (mean ± SD), contig N50 of 2.8 ± 1.1 kbp, and a mean of 185,000 ± 72,000 contigs per sample. These assembly metrics are consistent with those reported for human fecal metagenomes of comparable sequencing depth. Gene prediction was carried out using MetaGeneMark, and a non-redundant gene catalog was constructed by clustering predicted genes at 95% nucleotide identity using CD-HIT. Gene abundance in each sample was subsequently calculated by mapping clean reads back to this catalog using Bowtie2 (v2.4.2). Taxonomic annotation was achieved by aligning predicted genes against the NCBI NR database using DIAMOND (v2.0.15) with an e-value threshold of 1 × 10^-5^.

#### 2.4.4 Microbial community analysis

Alpha diversity was assessed using the Simpson index, Shannon index, and Observed Species richness. Beta diversity was evaluated using Bray–Curtis dissimilarity and visualized via principal coordinates analysis (PCoA). Group differences in community structure were tested using PERMANOVA with 999 permutations. Prior to differential abundance analysis, low-prevalence features were filtered by retaining only those species present at a relative abundance ≥0.01% in at least 20% of samples in either the ARFC or HC group.

Differentially abundant taxa were identified using a consensus-based approach that accounts for the compositional nature of microbiome data. Raw taxonomic counts were transformed using the centered log-ratio (CLR) transformation with a pseudocount of 0.5, and differential abundance was assessed using the Wilcoxon rank-sum test with Benjamini–Hochberg FDR correction. To complement this, Analysis of Compositions of Microbiomes with Bias Correction (ANCOM-BC) was applied using the R package ANCOMBC v2.0.1. Taxa were considered differentially abundant if they met the following criteria: (i) ANCOM-BC adjusted P < 0.05; (ii) Wilcoxon rank-sum test on CLR-transformed data FDR < 0.05; and (iii) Linear Discriminant Analysis Effect Size (LEfSe) LDA score > 2.

### 2.5 Targeted metabolomic profiling of amino acids and related metabolites

Given the established roles of amino acids as both microbial substrates and key immunomodulators, we focused our metabolomic investigation on this specific metabolite class to deeply characterize potential functional perturbations in ARFC.

#### 2.5.1 Sample preparation

Approximately 10 mg of lyophilized fecal powder was weighed and homogenized with two steel beads in 1000 µL of pre-cooled 90% methanol aqueous solution using a FastPrep-24 5G homogenizer (MP Biomedicals, USA) at 50 Hz for 6 minutes at -10 °C. After centrifugation at 14,000 × g for 20 min at 4 °C, 10 µL of the supernatant was transferred to a new tube and dried under a gentle stream of nitrogen. The residue was reconstituted in 50 µL of 50% acetonitrile in water and vortexed for 1 min. To account for matrix effects and variability in sample processing and ionization efficiency, a mixture of stable isotope-labeled internal standards (SIL-IS), including L-glutamine-^13^C_5_,^15^N_2_, L-alanine-^13^C_3_,^15^N, L-valine-d_8_, L-phenylalanine-^13^C^15^N, L-methionine-^13^C_5_,^15^N, and kynurenine-d_4_, was spiked into each sample at a fixed concentration prior to derivatization. Internal standards were selected to chemically represent each major amino acid class and to co-elute with their corresponding endogenous analytes, thereby enabling robust correction for technical variability. Derivatization was performed by adding 30 µL of 10 mg/mL dansyl chloride in acetone and 40 µL of 0.5 M sodium carbonate-bicarbonate buffer (pH 9.0). The mixture was vortexed and incubated at 60 °C in the dark for 30 min. The reaction was terminated by adding 10 µL of 0.25 M NaOH, followed by another 10 min incubation at 60 °C. Finally, 70 µL of 10% formic acid was added to acidify the mixture. After centrifugation at 14,000 × g for 15 min at 4 °C, the supernatant was transferred to an injection vial for LC-MS/MS analysis.

#### 2.5.2 LC-MS/MS analysis

Metabolite separation and detection were performed using an ExionLC AD liquid chromatography system coupled with a QTRAP^®^ 6500+ mass spectrometer (SCIEX, USA). Chromatographic separation was achieved on a Waters HSST3 column (2.1 × 150 mm, 1.8 µm) maintained at 40 °C. The mobile phase consisted of 0.1% formic acid in water (A) and 0.1% formic acid in methanol (B), with a flow rate of 0.35 mL/min. The gradient elution program was as follows: 0–2 min, 25% B; 2–3 min, 25–35% B; 3–8 min, 35–37% B; 8–8.5 min, 37–48% B; 8.5–11 min, 48% B; 11–14 min, 48–100% B; 14–15 min, 100% B. The mass spectrometer was operated in positive ion mode with an ion source temperature of 350 °C and a spray voltage of +5000 V. Data were acquired in multiple reaction monitoring (MRM) mode.

#### 2.5.3 Quality control and data normalization

A pooled quality control (PQC) sample, prepared by combining equal aliquots of all fecal extracts, was injected every ten study samples to monitor instrument stability. Calibration curves constructed using authentic standards exhibited R^2^ > 0.99 across a linear dynamic range of 0.01–10 μM. The lower limit of quantification was defined as the concentration at which the signal-to-noise ratio exceeded 10:1 with a coefficient of variation (CV) < 20%. Metabolite peak areas were normalized to corresponding stable isotope-labeled internal standards; for metabolites lacking a direct isotopic analog, the internal standard with the closest retention time was used. Batch effects were further adjusted using the ComBat algorithm (R package sva). The median CV across PQC injections was 8.7% (IQR: 5.2–12.4%), confirming acceptable reproducibility.

#### 2.5.4 Data processing and pathway analysis

Raw MS data were processed using SCIEX OS software for peak picking, integration, and quantification. Target amino acids and related metabolites were identified and relatively quantified by matching retention times and characteristic ion transitions against an in-house library of authentic standards. Orthogonal Partial Least Squares-Discriminant Analysis (OPLS-DA) was performed using SIMCA-P software (v14.1, Umetrics, Sweden) to identify metabolic signatures discriminating ARFC from HC children. Prior to modeling, metabolite data were Pareto-scaled to reduce the influence of high-abundance features while preserving the relative variance structure. Model validity and robustness were assessed using seven-fold cross-validation, yielding model quality parameters R^2^Y (cumulative explained variation) and Q^2^Y (cumulative predicted variation). A Q^2^Y value > 0.5 was considered indicative of acceptable predictive ability. To guard against model overfitting, a permutation test was conducted with 200 random permutations of the class labels. The model was deemed statistically valid only if the Q^2^Y values derived from permuted data were consistently lower than the Q^2^Y of the true model and if the regression line of permuted Q^2^Y values intersected the y-axis at ≤ 0.05. Differential metabolites were screened based on a variable importance in projection (VIP) score > 1.0 and an FDR-adjusted p-value < 0.05 (Benjamini-Hochberg method) derived from the Wilcoxon rank-sum test. Enrichment analysis of KEGG pathways for the differential metabolites was conducted using the MetaboAnalyst 5.0 online platform, with pathway significance assessed via hypergeometric testing and FDR correction (Pang et al., 2021).

### 2.6 Statistical analysis

All statistical analyzes were performed in R (v4.2.0). Continuous variables are presented as mean ± standard deviation (SD) or median (interquartile range) and compared using independent samples t−test or Mann–Whitney U test, as appropriate. Categorical variables are presented as frequencies (percentages) and compared using Chi−square or Fisher’s exact test. Spearman’s rank correlation was used to assess pairwise associations between microbial taxa, metabolites, and clinical parameters. To control for multiple comparisons, the Benjamini–Hochberg false discovery rate (FDR) correction was applied. A two−sided P < 0.05 or FDR < 0.05 was considered statistically significant. Effect sizes—including PERMANOVA R^2^ values, Spearman’s correlation coefficients (r), log_2_ fold−changes (Log_2_FC), and standardized mean differences (SMD) with 95% confidence intervals—are reported alongside P−values.

Diagnostic performance of the combined microbial–metabolic model was evaluated using receiver operating characteristic (ROC) curve analysis. To mitigate overfitting given the modest sample size (n = 35), a leave−one−out cross−validation (LOOCV) framework with L2−regularized (ridge) logistic regression was employed. Feature selection was performed using a Random Forest algorithm (mean decrease in accuracy > 0.3), and the top five discriminative features were retained for the cross−validated model. The area under the ROC curve (AUC) was calculated from aggregated LOOCV predictions, with 95% confidence intervals estimated using DeLong’s method. Sensitivity and specificity were determined at the optimal threshold defined by Youden’s index.

## 3 Results

### 3.1 Gut microbiota diversity analysis

#### 3.1.1 Altered beta diversity in children with ARFC

**Figure 1:**
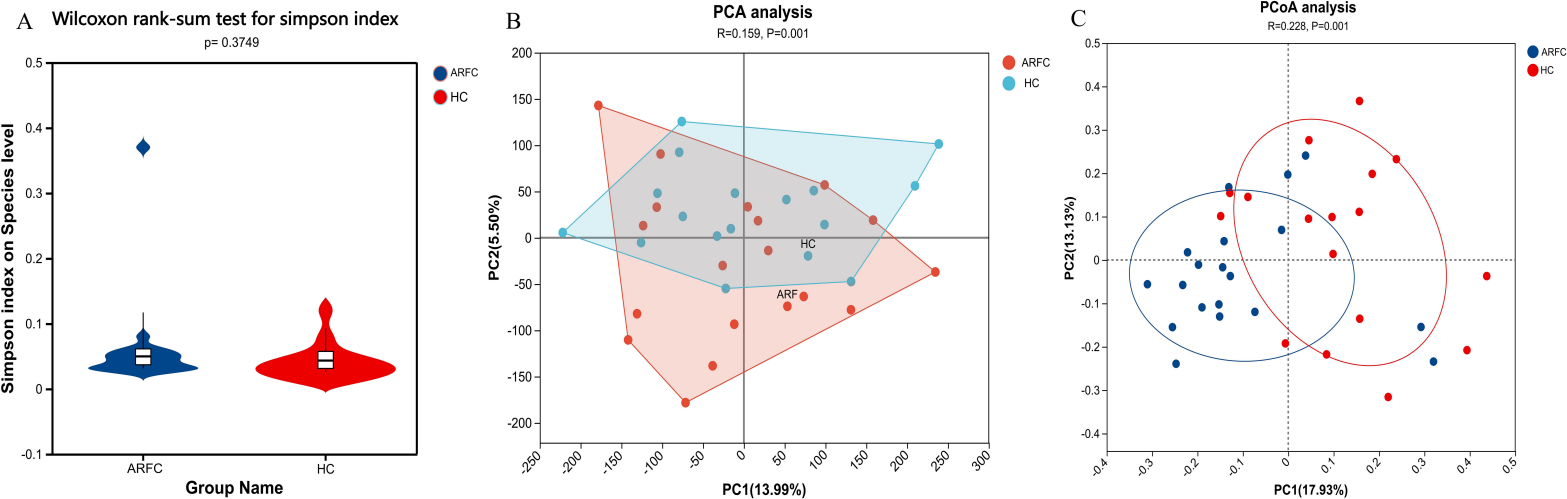
Gut microbiota alpha and beta diversity in ARFC and HC children. **(A)** Simpson index (species level), a measure of community evenness and dominance, showing no significant difference between groups (ns, *P* > 0.05, Mann-Whitney U test). Analyses of the Shannon index and Observed Species richness similarly revealed no significant differences (see **Supplementary Figure 1**). **(B)** Principal Component Analysis (PCA) plot based on Euclidean distance of species abundance. **(C)** Principal Coordinates Analysis (PCoA) plot based on Bray–Curtis dissimilarity. Both analyzes indicate significant separation of gut microbiota communities between ARFC and HC groups (PERMANOVA *P* = 0.001).

No statistically significant differences in alpha diversity were observed between the ARFC and HC groups across multiple complementary metrics. Specifically, comparisons revealed no significant differences in the Simpson index (*P* = 0.375; **Figure 1A**), Shannon index (*P* = 0.267; **Supplementary Figure 1**). In contrast, pronounced differences in beta diversity were observed. Both Principal Component Analysis (PCA) and Principal Coordinates Analysis (PCoA) based on Bray-Curtis dissimilarity revealed a statistically significant separation of the gut microbiota communities between groups (PCA: R^2^ = 0.159, *P* = 0.001; PCoA: R^2^ = 0.228, *P* = 0.001; **Figures 1B, C**).

#### 3.1.2 Differences in gut microbiota composition

**Figure 2:**
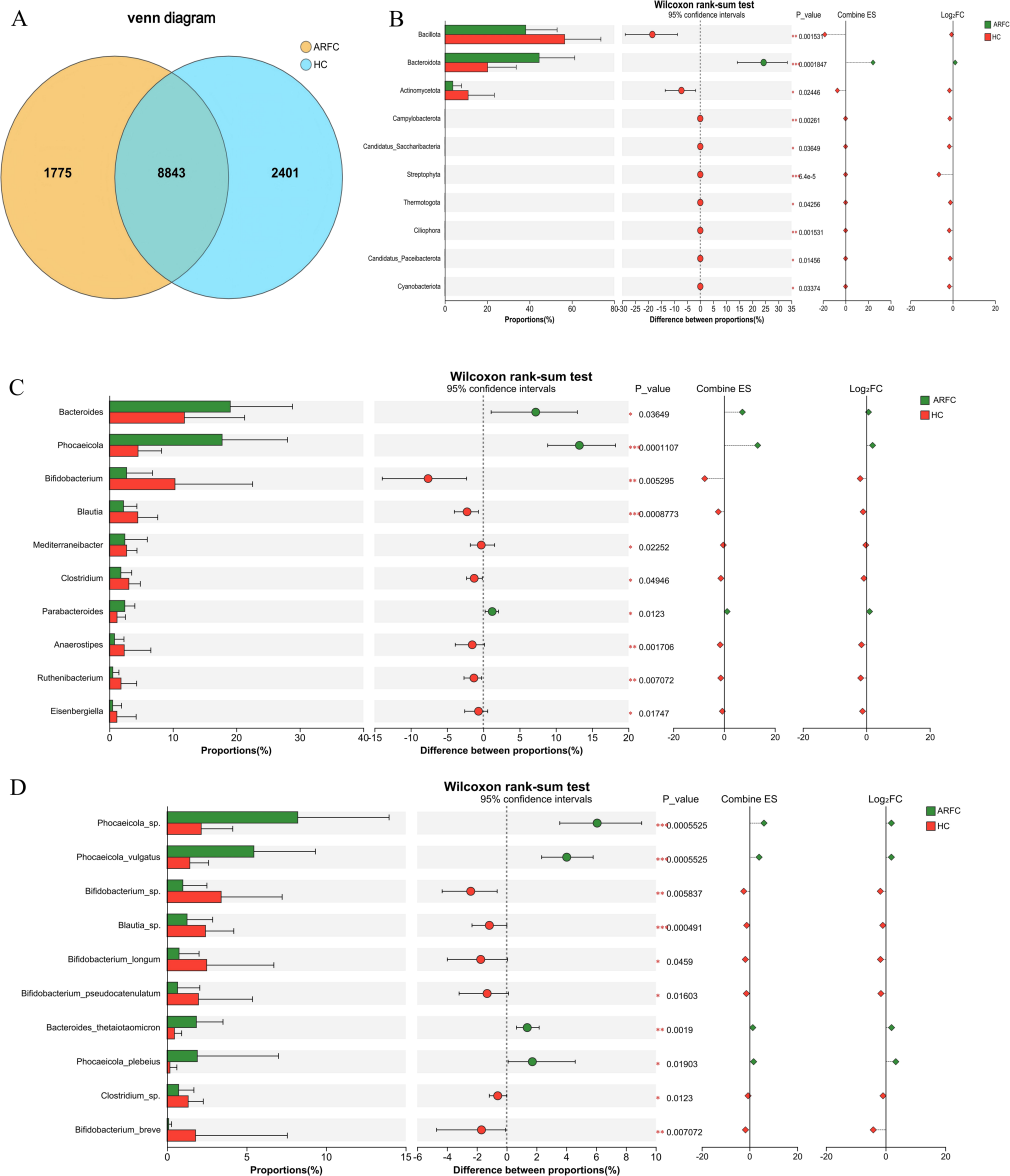
Comparative analysis of gut microbiota composition between ARFC and HC children. **(A)** Venn diagram showing shared and unique bacterial species between groups (ARFC: 1775 unique; HC: 2401 unique; shared: 8843). **(B)** Bar plot of differentially abundant phyla. ARFC group exhibited higher Bacteroidota and lower Bacillota and Streptophyta (Wilcoxon rank-sum test, FDR < 0.05). **(C)** LEfSe analysis identifying discriminative bacterial genera. *Bacteroides* and *Phocaeicola* were enriched in ARFC, whereas *Bifidobacterium* and *Blautia* were enriched in HC (LDA > 2, FDR < 0.05). **(D)** Heatmap of the top 10 differentially abundant bacterial species (Wilcoxon rank-sum test, FDR < 0.05).

The two groups shared 8843 species, with 1775 and 2401 species unique to ARFC and HC, respectively (**Figure 2A**). At the phylum level, the relative abundance of Bacteroidota was significantly higher in the ARFC group (*p* = 0.0001847), whereas Bacillota (*p* = 0.001531) and Streptophyta (*p* = 6.394E-5) were significantly enriched in the HC group (**Figure 2B**). LEfSe analysis identified several differentially abundant genera (LDA > 2, FDR < 0.05; **Figure 2C**; **Table 2**). *Bacteroides, Phocaeicola*, and *Parabacteroides* were significantly enriched in the ARFC group (all *P* < 0.05), while *Bifidobacterium* and *Blautia* were more abundant in the HC group (all *P* < 0.01; **Figure 2C**; detailed data in **Table 2**). At the species level, *Phocaeicola* sp. (*P* = 0.001), *Phocaeicola vulgatus* (*P* = 0.001), *Bacteroides thetaiotaomicron* (*P* = 0.002), and *Phocaeicola plebeius* (*P* = 0.019) were significantly more abundant in the ARFC group. In contrast, *Bifidobacterium* sp. (*P* = 0.006), *Blautia* sp. (*P* = 0.001), *Bifidobacterium longum* (*P* = 0.046), *Bifidobacterium pseudocatenulatum* (*P* = 0.016), *Clostridium* sp. (*P* = 0.012), and *Bifidobacterium breve* (*P* = 0.007) were significantly enriched in the HC group (**Figure 2D**; **Table 3**).

**Table 2:** The top 10 bacterial genera with the highest average abundance in two groups of children.

Name	ARFC-mean(%)	ARF-Sd(%)	HC-mean(%)	HC-Sd(%)	P-adjust
*Bacteroides*	19.047	9.806	11.827	9.480	0.037
*Phocaeicola*	17.758	10.309	4.514	3.704	0.000
*Bifidobacterium*	2.717	4.079	10.333	12.226	0.005
*Mediterraneibacter*	2.434	3.546	2.719	1.629	0.023
*Parabacteroides*	2.427	1.586	1.204	1.328	0.012
*Blautia*	2.231	2.099	4.474	3.139	0.001
*Clostridium*	1.809	1.704	3.068	1.810	0.049
*Streptococcus*	0.902	3.539	0.707	0.504	0.000
*Anaerostipes*	0.813	1.484	2.342	4.193	0.002
*Ruthenibacterium*	0.534	0.969	1.831	2.462	0.007

**Table 3:** The top 10 bacterial species with the highest average abundance in two groups of children .

Name	ARFC-mean(%)	ARF-Sd(%)	HC-mean(%)	HC-Sd(%)	P-adjust
*Phocaeicola* sp.	8.215	5.743	2.149	1.982	0.001
*Phocaeicola vulgatus*	5.457	3.870	1.430	1.180	0.001
*Bacteroides thetaiotaomicron*	1.845	1.670	0.466	0.451	0.0019
*Blautia* sp.	1.257	1.610	2.416	1.779	0.001
*Bifidobacterium* sp.	0.992	1.513	3.40	3.834	0.006
*Bifidobacterium longum*	0.753	1.259	2.491	4.222	0.046
*Clostridium* sp.	0.729	0.958	1.328	0.950	0.012
*Bifidobacterium pseudocatenulatum*	0.662	1.396	1.973	3.398	0.016
*Ruthenibacterium lactatiformans*	0.389	0.647	1.349	1.838	0.009
*Anaerostipes* sp.	0.358	0.582	0.993	1.637	0.003

### 3.2 Targeted metabolomic analysis of amino acids

#### 3.2.1 Comparative analysis of metabolic profiles

**Figure 3:**
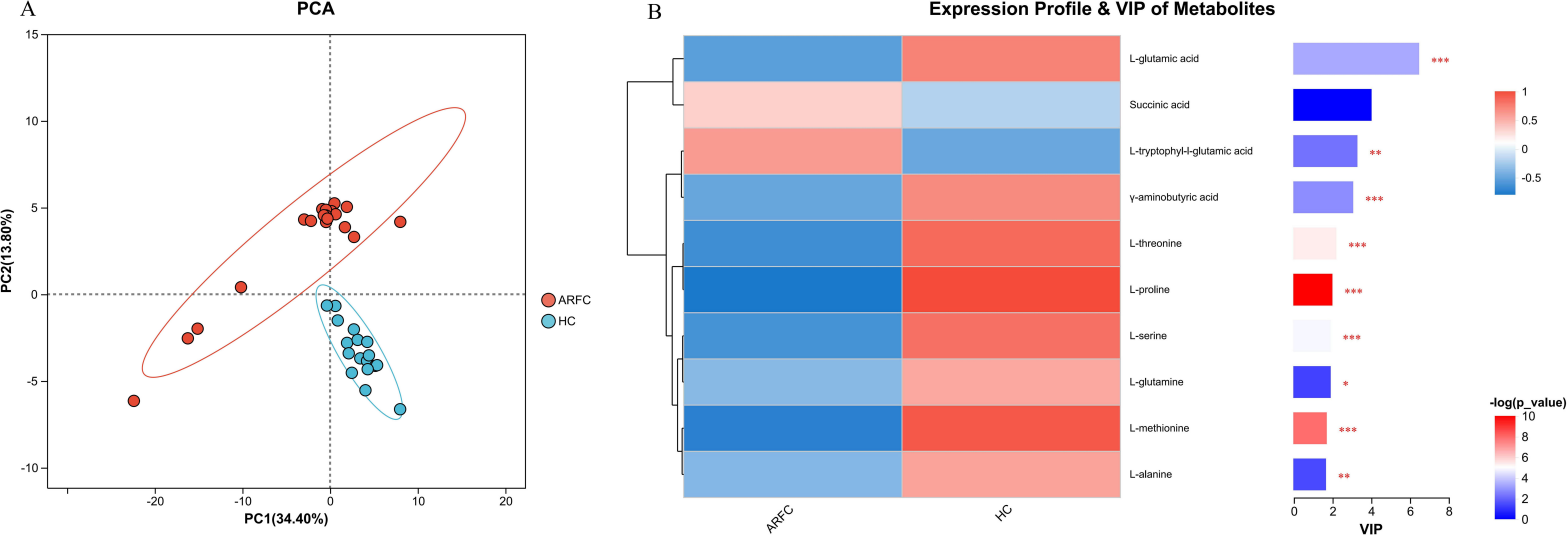
Amino acid-targeted metabolomic profiling in ARFC and HC children. **(A)** PCA score plot demonstrating distinct clustering between ARFC and HC groups based on fecal amino acid profiles. **(B)** Hierarchical clustering heatmap of the 50 differentially abundant metabolites (VIP > 1.0, FDR-adjusted *P* < 0.05). Columns represent samples; rows represent metabolites. Red and blue indicate up- and downregulation, respectively.

Principal Component Analysis (PCA) of the metabolomic data showed clear separation between the ARFC and HC groups (**Figure 3A**). Supervised Orthogonal Partial Least Squares-Discriminant Analysis (OPLS-DA) was employed to delineate metabolic signatures discriminating ARFC from HC children. The derived model exhibited robust explanatory and predictive capacity, with a cumulative R^2^Y of 0.91 and a cumulative Q^2^Y of 0.73, indicating that 91% of the variation in the metabolite data could be explained by the model and that it possessed substantial predictive accuracy. A 200-iteration permutation test confirmed the model’s statistical validity, as all permuted Q^2^Y values were lower than the true Q^2^Y value, and the regression line of permuted Q^2^Y values intersected the y-axis at a negative value of -0.41. This model identified 50 metabolites that significantly differed between groups (VIP > 1.0, FDR-adjusted *P* < 0.05), of which 44 were upregulated and 6 were downregulated in the ARFC group. A hierarchical clustering heatmap of these differential metabolites is presented in **Figure 3B**, illustrating distinct clustering by disease status.

Notably, L-glutamic acid showed the highest VIP score (6.4) and was significantly downregulated in ARFC. Multiple other amino acids were also markedly reduced in ARFC, including L-proline (VIP = 2.0), L-threonine (VIP = 2.2), L-serine (VIP = 1.9), and L-methionine (VIP = 1.7). Furthermore, the level of γ-aminobutyric acid (GABA), a critical neuro-immunomodulatory metabolite, was significantly lower in ARFC children (VIP = 3.0). In contrast, L-tryptophyl-L-glutamic acid was significantly elevated in the ARFC group (VIP = 3.3). This analysis underscores L-glutamic acid and GABA as central, highly discriminative features within the perturbed amino acid metabolome of ARFC.

**Table 4** lists the top 10 fecal metabolites that differed significantly between the ARFC and HC groups, ranked primarily by statistical significance (adjusted P-value and fold-change magnitude) rather than by VIP scores from the OPLS-DA model. Notably, this list was dominated by aromatic and branched-chain amino acids and their derivatives, indicating a profound disruption of amino acid homeostasis in ARFC children. Several key amino acids were significantly downregulated in the ARFC group, including L-glutamic acid, L-proline, L-glutamine, L-alanine, L-valine, L-methionine, and 4-acetamidobutyric acid. Conversely, a subset of metabolites was upregulated in ARFC, such as L-tryptophyl-L-glutamic acid, L-phenylalanine, and kynurenine. This distinct profile of differentially abundant amino acids and related compounds highlights specific metabolic perturbations that may be linked to the pathophysiology of ARFC.

**Table 4:** Top 10 gut microbiota metabolites with significant differences.

Metabolite	ARFC- mean	HC-mean	P-adjust	Log 2FC	Adjust direction
L-glutamic acid	8852005	15090480	0.002	-0.7695	down
L-tryptophyl-L-glutamic acid	2074823.8	77353.1	0.007	4.4536	up
L-proline	167527.3	666273.5	0.000	-1.992	down
L-glutamine	1083987	2323168	0.031	-1.0996	down
L-alanine	995752.4	1599828	0.021	-0.6841	down
L-valine	598173.1	1018895	0.004	-0.7685	down
L-phenylalanine	408229.3	240964.7	0.041	0.7602	up
L-methionine	8111.8	387413.5	0.000	-5.5776	down
4-acetamidobutyric acid	7087.1	20978.6	0.003	-1.5658	down
Kynurenine	1238.5	223.6	0.002	2.4690	up

#### 3.2.2 Enrichment of amino acid metabolic pathways

**Figure 4:**
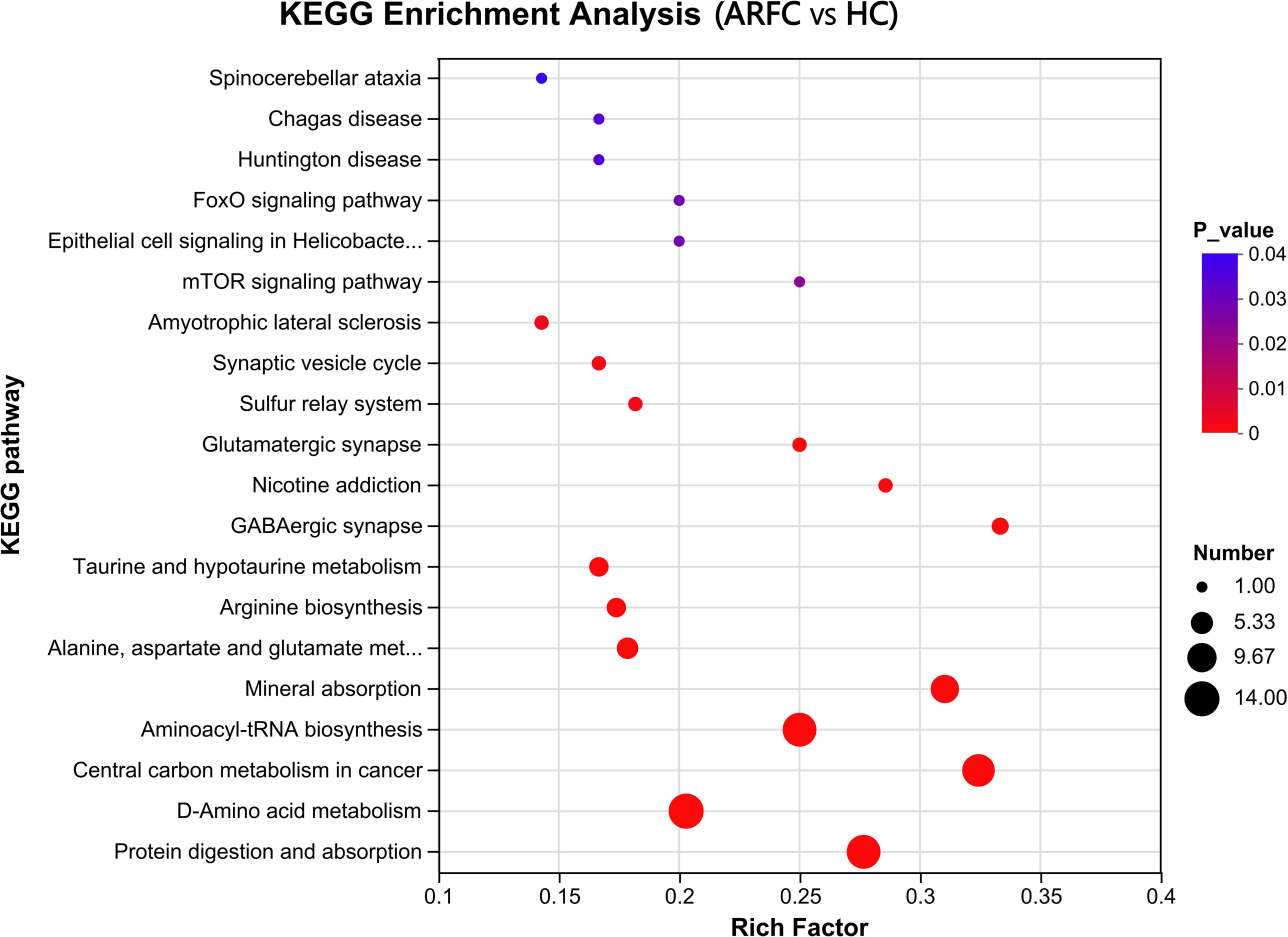
Enrichment analysis of metabolic pathways based on differentially abundant fecal metabolites. Bubble chart illustrating KEGG pathway enrichment results. Bubble size corresponds to the number of mapped metabolites; color intensity represents –log_10_(P-value). Significantly enriched pathways (adjusted P < 0.05) include mTOR signaling, FoxO signaling, GABAergic synapse, and Glutamatergic synapse.

KEGG enrichment analysis revealed significant dysregulation of several pathways in ARFC (**Figure 4**). These included signaling pathways (mTOR and FoxO), neurotransmitter systems (GABAergic and glutamatergic synapses), and amino acid metabolism pathways (e.g., alanine, aspartate, and glutamate metabolism).

### 3.3 Integrated analysis of gut microbiota and metabolites

#### 3.3.1 Interactions between gut microbiota and metabolites

**Figure 5:**
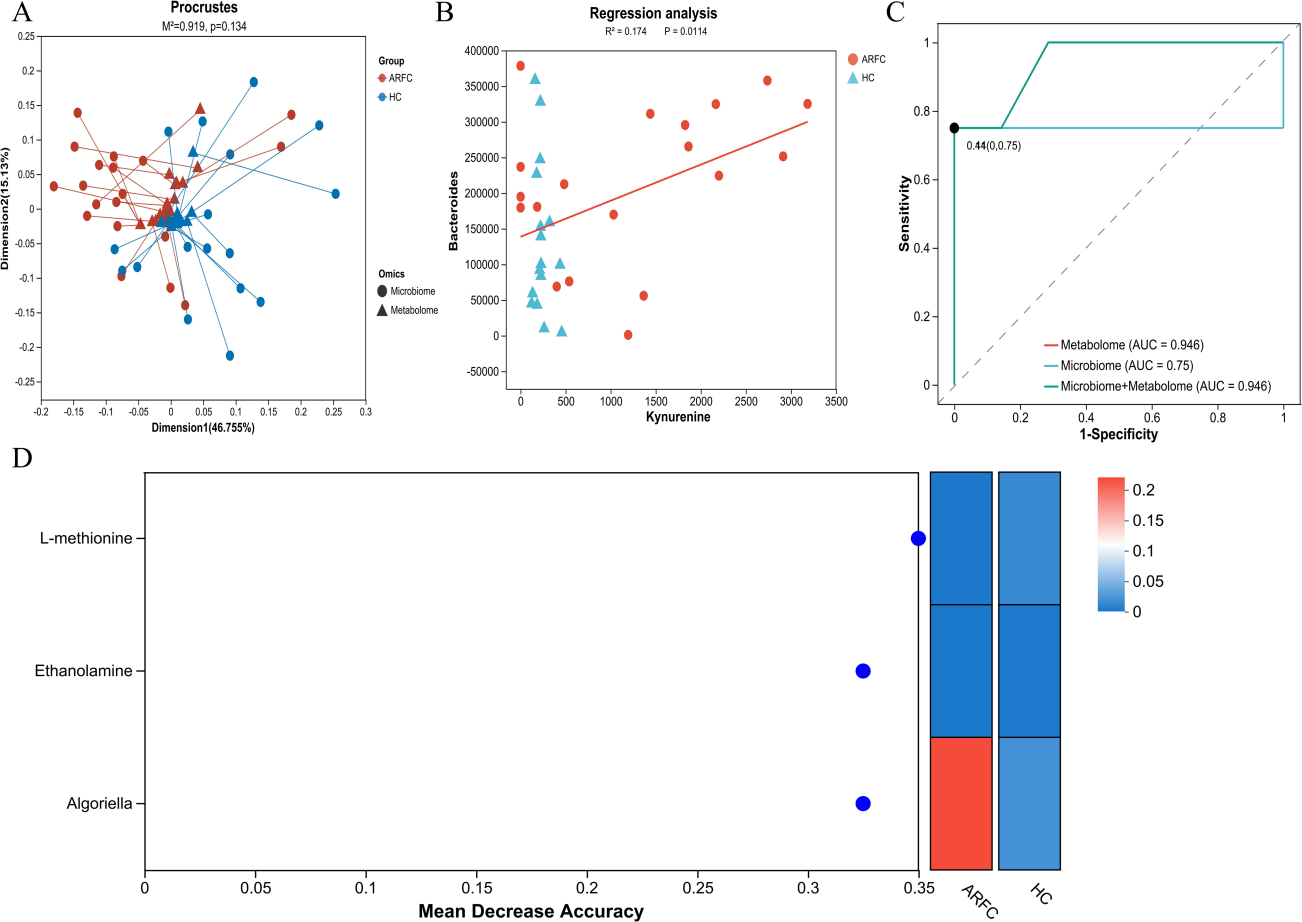
Integrated analysis of gut microbiota and metabolites, and diagnostic model performance. **(A)** Procrustes analysis comparing overall structure of microbiota and metabolome profiles (M^2^ = 0.919, *P* = 0.134). **(B)** Scatter plots showing significant linear correlations between selected microbial genera and metabolite levels (e.g., *Bacteroides* vs. kynurenine, R^2^ = 0.174, *P* = 0.0114). **(C)** Receiver operating characteristic (ROC) curve illustrating the discriminative performance of the combined microbial--metabolic diagnostic model. The area under the curve (AUC) of 0.946 (95% CI: 0.86-1.00) was derived from a leave-one-out cross-validation (LOOCV) framework using L2-regularized logistic regression, providing a robust estimate of model generalizability. **(D)** Variable importance plot from Random Forest analysis ranking the top discriminative features for ARFC vs. HC classification.

Procrustes analysis showed no significant overall structure congruence between microbiota and metabolome profiles (M^2^ = 0.919, *P* = 0.134; **Figure 5A**). However, the relatively high M^2^ value suggests a degree of difference between the two datasets after optimal transformation.

Moreover, linear regression analysis revealed significant covariations between specific gut microbial genera and fecal metabolites (**Figure 5B**). The highlighted positive correlation between the genus *Bacteroides*—a taxon enriched in ARFC—and kynurenine, a key metabolite in tryptophan metabolism and immune regulation, strongly suggests that the observed metabolomic alterations are, at least in part, a functional output of the dysbiotic microbiota.

A combined model integrating key microbial and metabolic features, evaluated under a leave-one-out cross-validation (LOOCV) framework with L2-regularized logistic regression, demonstrated strong discriminative performance in this cohort. The cross-validated area under the ROC curve (AUC) was 0.946 (95% CI: 0.86–1.00), with sensitivity of 0.89 and specificity of 0.94 at the optimal probability threshold (Youden’s index) (**Figure 5C**). The unregularized, full-feature model yielded an AUC of 1.0 (95% CI: 1.0–1.0) in the training set; however, this value likely reflects overfitting given the modest sample size, and we therefore emphasize the cross-validated estimate (AUC = 0.946) as the more realistic indicator of model performance. Feature importance was evaluated using a Random Forest algorithm to rank the relative contributions of key microbial and metabolic features in distinguishing ARFC from HC. The results, visualized in a variable importance scatter plot (**Figure 5D**), identified the amino acid L-methionine as the top-ranking feature, exhibiting the highest values for both the Mean Decrease in Accuracy (0.35) and the Gini Index (0.35). The gut microbial genus Algoriella also demonstrated high importance, with equivalent Mean Decrease in Gini (0.35) and a slightly lower Mean Decrease in Accuracy (0.325). The metabolite Ethanolamine was ranked as the third most important feature (Mean Decrease in Accuracy = 0.325, Mean Decrease in Gini = 0.325). This ranking highlights that a combination of a specific amino acid metabolite (L-methionine) and a bacterial genus (Algoriella) were the most discriminative features within the integrated model, underscoring their potential roles in the pathophysiology of ARFC.

#### 3.3.2 Correlations between gut microbiota and allergic parameters

**Figure 6:**
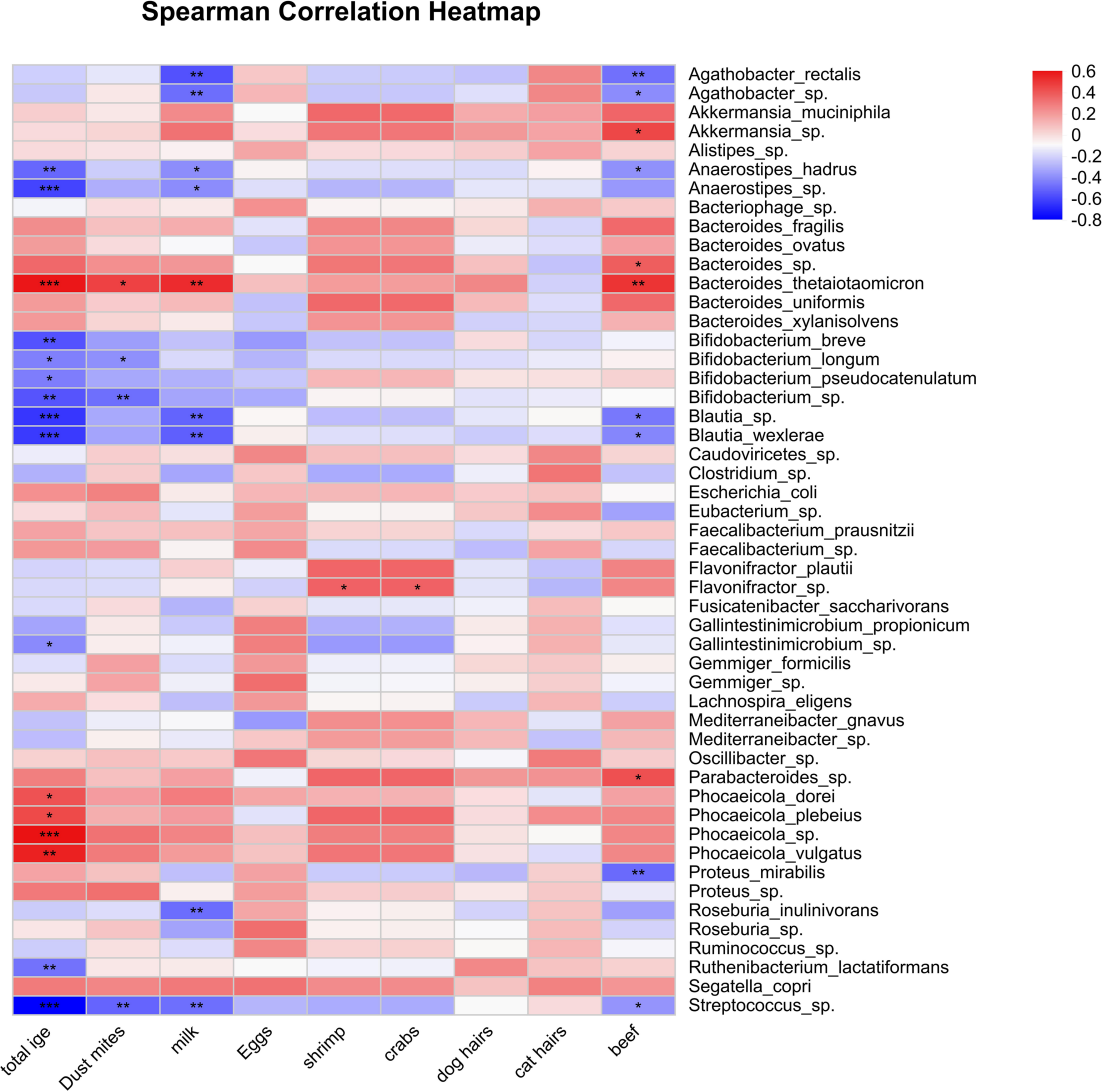
Correlations between gut microbiota species and clinical allergy parameters in children. Heatmap of Spearman’s correlation coefficients between relative abundances of 46 bacterial species (rows) and 9 clinical IgE measures (columns). Red and blue denote positive and negative correlations, respectively; asterisks indicate statistical significance (FDR < 0.05). Key associations include positive correlations of *Bacteroides thetaiotaomicron* with dust mite-specific and total IgE, and negative correlations of *Bifidobacterium breve* with both IgE measures.

Significant correlations were observed between specific bacterial species and IgE levels (**Figure 6A**). For example, *Bacteroides thetaiotaomicron* positively correlated with dust mite-specific IgE (r = 0.423) and total IgE (r = 0.545), whereas *Bifidobacterium breve* showed negative correlations (dust mite-IgE: r = -0.316; total IgE: r = -0.505).

Significant correlations were observed between specific bacterial species and clinical allergy parameters (**Figure 6**). For instance, the relative abundance of *Bacteroides thetaiotaomicron* showed a significant positive correlation with both dust mite-specific IgE (r=0.423) and total IgE (r=0.545). Conversely, species such as *Bifidobacterium breve* exhibited negative correlations with dust mite-specific IgE (r=-0.316) and total IgE (r=-0.505). Significant associations were also found with various food and inhalant allergen-specific IgE levels (see **Supplementary Table 2** for complete data).

## 4 Discussion

This pioneering study integrates metagenomic sequencing and amino acid-targeted metabolomics to delineate the distinct gut ecosystem in children with ARFC. We uncover a synergistic dysbiosis characterized by an expansion of mucolytic Bacteroidota alongside depletion of beneficial, SCFA-producing Bacillota, coupled with extensive perturbations in fecal amino acid profiles. Crucially, these microbial and metabolic shifts are intricately linked, forming a correlated network with clinical allergy markers, thereby providing a novel multi-omics perspective on the “gut-lung-enteric” axis in this comorbidity.

### 4.1 Gut dysbiosis in ARFC: a pro-inflammatory and barrier-compromising phenotype

Our metagenomic analysis reveals a distinct gut microbiota structure in ARFC children, characterized by an enrichment of Bacteroidota (particularly genera like *Bacteroides* and *Phocaeicola*) and a depletion of Bacillota (including *Bifidobacterium* and *Blautia*) (**Figures 2B, C**). This dysbiotic signature aligns with profiles observed in other allergic conditions (De Filippis et al., 2021; Hoskinson et al., 2023). However, the interpretation of these phylum-level shifts requires nuance, as both Bacteroidota and Bacillota encompass functionally diverse taxa whose contributions to host health are highly context-dependent. For instance, Bacteroidota includes both beneficial short-chain fatty acid (SCFA)-producing species and mucolytic organisms that may compromise barrier integrity, while Bacillota comprises immunomodulatory commensals alongside potential pathobionts. Notably, recent work specifically characterizing AR-FC comorbidity has demonstrated depletion of the butyrate-producing species *Faecalibacterium prausnitzii* and a paradoxical enrichment of *Bifidobacterium*—the latter potentially reflecting a compensatory immunomodulatory response to persistent allergic inflammation (Yang et al., 2025). Furthermore, the elevated relative abundance of *Bacteroides thetaiotaomicron* observed in our cohort should not be interpreted unequivocally as pathogenic; experimental evidence has demonstrated that oral administration of *B. thetaiotaomicron* ameliorates ovalbumin-induced allergic airway inflammation in mice via activation of ICOS^+^ regulatory T cells and suppression of Th2 cytokine production, without promoting a Th1 response (Pang et al., 2021). Collectively, these observations underscore that the functional consequences of ARFC-associated dysbiosis likely depend on the specific species and strains involved, their metabolic activities, and the broader ecological context—a complexity that transcends simple phylum-level characterizations.

The observed enrichment of mucin-degrading species such as *B. thetaiotaomicron* in ARFC children may be associated with compromised intestinal barrier integrity, as supported by studies linking its mucolytic activity to barrier dysfunction (Ndeh and Gilbert, 2018; Wang et al., 2024). This could potentially facilitate the translocation of microbial products and dietary antigens, possibly priming a systemic inflammatory state and a Th2-polarized immune response—a plausible but still hypothetical link to airway inflammation in AR (Ndeh and Gilbert, 2018; Arifuzzaman et al., 2024). Conversely, the depletion of beneficial genera such as *Bifidobacterium* and *Blautia* is consistent with a potential deficit in short-chain fatty acid (SCFA) production. SCFAs, including butyrate, are well-established immunomodulators that support intestinal barrier integrity and facilitate regulatory T (Treg) cell differentiation (Arpaia et al., 2013; Parada Venegas et al., 2019). It is plausible that the diminished abundance of SCFA-producing taxa observed in ARFC may be accompanied by reduced barrier-strengthening metabolites and impaired Treg induction—a process in which specific commensal clostridia strains have been shown to participate (Atarashi et al., 2013). Furthermore, the depletion of anti-inflammatory species such as *Faecalibacterium prausnitzii*—known to mitigate low-grade inflammation and support epithelial health (Martín et al., 2015)—may further exacerbate the pro-inflammatory milieu in the ARFC gut.

### 4.2 Amino acid metabolic dysregulation: linking microbial ecology to host immunometabolism

Our targeted metabolomics data extend the taxonomic findings by revealing widespread disturbances in amino acid metabolism, functionally linking gut dysbiosis to host immunometabolism (**Figure 3**). It should be noted that this analysis was intentionally restricted to amino acids and related metabolites; other important classes of gut microbiota-derived compounds—including short-chain fatty acids (SCFAs) and bile acids—were not assessed in the present study. We observed significant downregulation of key immunomodulatory metabolites, including L-glutamine (crucial for lymphocyte function and gut barrier integrity (Cruzat et al., 2018)) and γ-aminobutyric acid (GABA, a microbiota-derived neuro-immunomodulator (Qu et al., 2024)). This suggests a metabolic milieu unfavorable for immune homeostasis. Pathway enrichment analysis further implicated critical nutrient-sensing and immune-regulatory pathways. The significant enrichment of the mTOR and FoxO signaling pathways (**Figure 4**) is particularly salient. mTOR integrates amino acid availability to regulate T-cell fate; its overactivation is known to promote Th2 differentiation while suppressing Treg function (Pollizzi and Powell, 2014; Hou et al., 2020). The reduction in glutamine and GABA levels observed in ARFC suggests a potential source of aberrant nutritional signals that could influence mTOR activity in immune cells. Concurrently, the association with FoxO pathway dysregulation—a pathway essential for Treg stability (Kerdiles et al., 2010)—raises the possibility of further immune imbalance. Collectively, within the constraints of our amino acid-focused metabolomic approach, these correlative findings support a model in which amino acid metabolism may serve as a functional intermediary linking gut microbial composition to immunometabolic perturbations in ARFC.

### 4.3 A tripartite network: correlating microbiota, metabolites, and clinical allergy

The integrative analysis yielded a clinically pertinent interaction network, directly linking gut microbial ecology with systemic allergic responses. The observed strong positive correlations between ARFC-enriched *B. thetaiotaomicron* and both total and dust mite-specific IgE levels suggest a potential association between this bacterial species and allergic sensitization. The absence of significant global congruence between the microbiota and metabolome datasets in Procrustes analysis (M^2^ = 0.919, P = 0.134; **Figure 5A**) warrants careful interpretation and does not negate the existence of specific, biologically meaningful associations. Procrustes analysis evaluates the overall structural correspondence between two ordinations and is inherently conservative; it can fail to detect significant feature-level correlations when global data structures are complex or when only a subset of variables covaries (Deek et al., 2024). Furthermore, identical microbial communities can produce divergent metabolic profiles depending on environmental context, substrate availability, and host factors—a phenomenon that may obscure global congruence despite the presence of specific microbiota–metabolite interactions. In the present study, while global congruence was non-significant, we identified multiple significant pairwise correlations between individual microbial genera and metabolites (e.g., *Bacteroides* with kynurenine, R^2^ = 0.174; **Figure 5B**). This pattern—global discordance accompanied by local, feature-level associations—is consistent with the interpretation that specific microbial taxa, rather than the entire community, drive particular metabolic outputs, and that these discrete interactions may collectively contribute to the ARFC phenotype without manifesting as global omic congruence.

However, these correlational data do not establish causality and may reflect a shared underlying immune dysregulation, a notion supported by studies linking gut microbiome composition to host inflammatory cytokine production capacity (Schirmer et al., 2016; Zhang et al., 2025). Notably, the integration of metabolomic data provides additional correlative context for this microbial-immunological association. For example, the positive correlation between the genus *Bacteroides* and kynurenine (**Figure 5B**) suggests a potential link between dysbiosis and tryptophan metabolism—a pathway with recognized immunoregulatory functions in allergic disease (Cervantes-Barragan et al., 2017; Laurans et al., 2018). Other identified microbiota-metabolite associations further support that the perturbed metabolome is a functional output of the dysbiotic microbiota. The combined microbial-metabolic model demonstrated perfect discrimination in our cohort (AUC = 0.946; **Figure 5C**), highlighting its potential as a diagnostic tool. However, this exceptional performance likely reflects the limited sample size and requires validation in larger, independent cohorts to assess its generalizability and translational potential, a critical step emphasized in biomarker development studies (Hao et al., 2025).

### 4.4 Toward an integrative pathophysiological model

**Figure 7:**
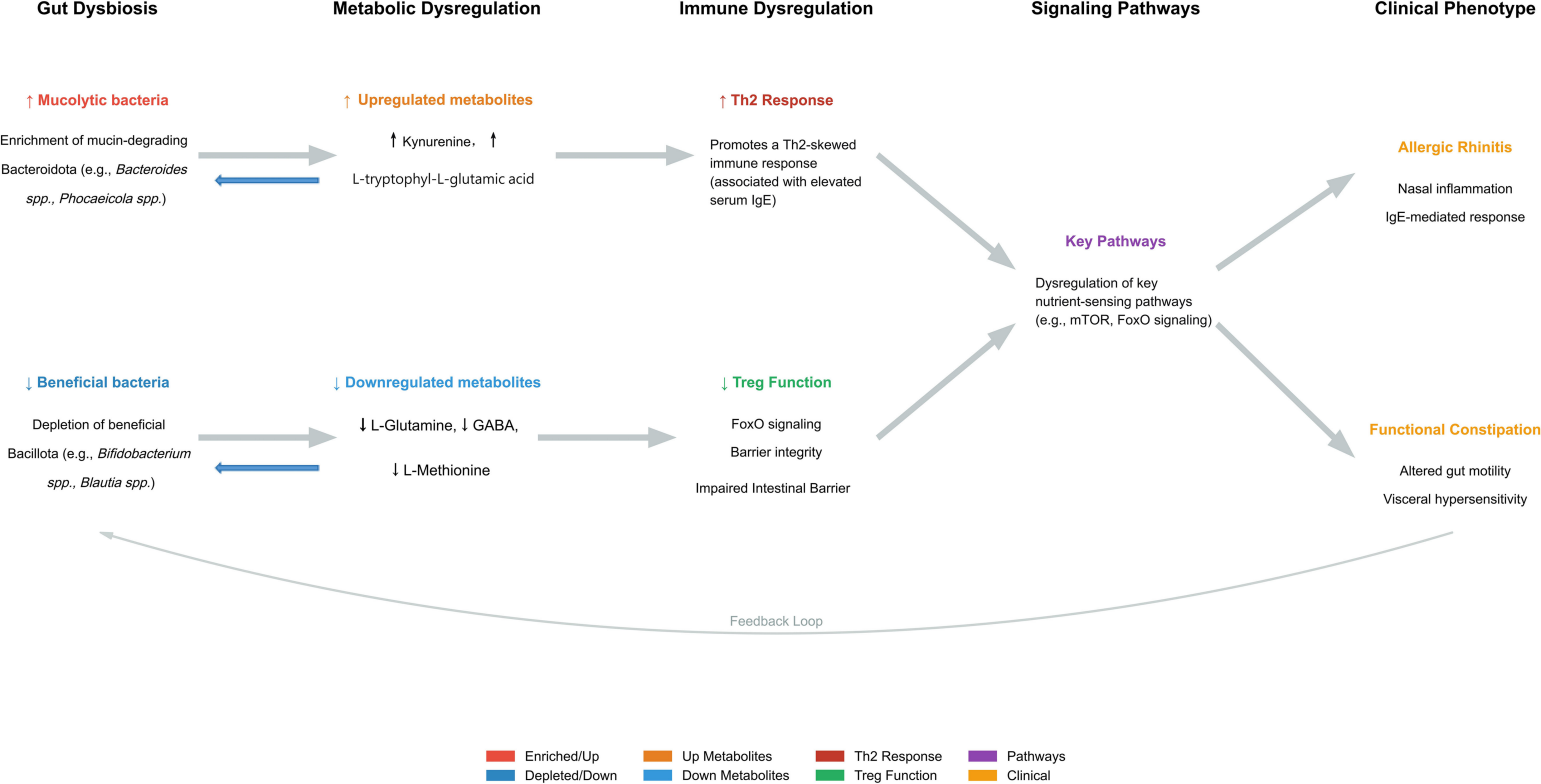
Proposed pathophysiological model of the “gut microbiota-metabolism-immunity” axis in ARFC. The schematic illustrates the hypothetical cascade linking gut dysbiosis to the comorbid allergic rhinitis and functional constipation (ARFC) phenotype. 1) Gut dysbiosis: ARFC children exhibit enrichment of mucin-degrading Bacteroidota (e.g., *Bacteroides thetaiotaomicron*) and depletion of beneficial, short-chain fatty acid (SCFA)-producing Bacillota (e.g., *Bifidobacterium* spp.). 2) Metabolic dysregulation: This dysbiotic profile is associated with altered fecal amino acid metabolism, characterized by reduced immunomodulatory metabolites (e.g., L-glutamine, GABA) and increased kynurenine. 3) Immune dysregulation: The metabolic shifts contribute to immune imbalance, promoting a Th2-polarized response (elevated IgE, IL-4/5/13) while impairing regulatory T (Treg) cell function and barrier integrity. 4) Signaling pathways: Key nutrient-sensing pathways (mTOR and FoxO) are dysregulated, further amplifying the pro-inflammatory state. 5) Clinical phenotype: The integrated dysregulation manifests as the dual symptoms of allergic rhinitis (upper airway inflammation) and functional constipation (altered gut motility). Arrows indicate proposed causal relationships and feedback loops. This integrative model provides a framework for understanding ARFC pathogenesis and identifies potential targets for microbiome-based interventions.

Based on the integrative correlative findings presented herein, we propose a hypothetical working model that may help conceptualize the relationships among gut dysbiosis, metabolic perturbations, and clinical features in ARFC (**Figure 7**). In this framework, the dysbiotic gut ecosystem—characterized by an increased relative abundance of mucolytic *B. thetaiotaomicron* and a decreased abundance of SCFA-producing *Bifidobacterium*—is associated with two potentially interrelated phenomena: alterations in intestinal barrier integrity and shifts in metabolic output. The latter include reduced levels of immunomodulatory metabolites (e.g., glutamine, GABA) and an altered amino acid profile that coincides with enrichment of nutrient-sensing pathways (mTOR/FoxO) identified in our KEGG analysis. The observed correlations between *B. thetaiotaomicron* and both IgE levels and kynurenine suggest that this species may warrant further investigation as a candidate contributor to the ARFC phenotype. Collectively, these associations are consistent with a Th2-skewed, IgE-high state that may involve both the respiratory mucosa and gut motility—the defining features of ARFC. This “gut microbiota-metabolism-immunity” axis, elucidated by our correlation networks, provides a holistic framework for understanding this common comorbidity. This breach in intestinal barrier integrity may facilitate the translocation of microbial products such as lipopolysaccharide (LPS), which can engage immune receptors and amplify systemic inflammation, a mechanism implicated in gut–organ axes in inflammatory conditions (Candelli et al., 2021).

### 4.5 Therapeutic implications: probiotics as a microbiome-targeted strategy

The dysbiotic signature delineated herein—characterized by depletion of beneficial Bacillota (*Bifidobacterium, Blautia*) and deficient immunomodulatory amino acid production—provides a compelling rationale for microbiome-targeted interventions in ARFC. Probiotic supplementation has emerged as a promising therapeutic avenue for pediatric allergic disease. A recent systematic review and network meta-analysis of 18 randomized controlled trials encompassing 1,789 pediatric patients demonstrated that probiotics significantly reduce Total Nasal Symptom Scores (SMD = −0.85, 95% CI [−1.25, −0.44]) and improve disease-specific quality of life, with multi-strain formulations conferring the greatest benefit (Li et al., 2026). Notably, serum total IgE levels remained unchanged (SMD = −0.34, *P* = 0.18), suggesting that clinical efficacy is mediated primarily through local mucosal immunomodulation rather than systemic IgE suppression—a finding consistent with the gut-resident immune dysregulation observed in our cohort.

Several recently characterized probiotic strains exemplify the potential for rational selection of candidates to address ARFC-associated dysbiosis. Genomic and functional analyzes of *Lactiplantibacillus plantarum MB685*, isolated from fermented broccoli, have confirmed its robust bile tolerance, auto-aggregation capacity, and the absence of transferable antibiotic resistance or virulence determinants (Irfan et al., 2026). Similarly, Pediococcus pentosaceus SPARC2 exhibits favorable enzymatic activities and antagonistic effects against pathogens, and its oral administration in BALB/c mice enhanced beneficial bacterial populations without inducing colonic inflammation (Butt et al., 2025). These exemplars highlight the expanding repertoire of genomically vetted probiotics that could be deployed to replenish depleted Bacillota populations. The immunomodulatory potential of L. plantarum is further supported by evidence that oral administration of strain NR16 ameliorates murine allergic rhinitis by restoring Th2/Th1 balance and reducing nasal mucosal Th2 cytokine production (Yang et al., 2022).

The translational relevance of this approach to the AR-FC comorbidity is reinforced by a recent synbiotic intervention study in preschool children with AR-FC (Yang et al., 2025). Following a three-month regimen of multi-strain probiotics combined with dietary fiber, the abundance of *Faecalibacterium*—a key short-chain fatty acid-producing genus—increased by 54.8%, and constipation symptoms improved. Importantly, the observed 85.2% reduction in *Bifidobacterium* abundance underscores the necessity for strain-specific selection, as non-specific formulations may inadvertently fail to restore all depleted beneficial taxa.

Collectively, these findings advocate for future interventional studies in ARFC to prioritize rationally designed probiotic or synbiotic formulations that not only replenish SCFA-producing taxa but also incorporate strains with demonstrated immunomodulatory capacity. The multi-omics framework established herein may facilitate the targeted identification and evaluation of such next-generation biotherapeutics, with the goal of assessing their potential to modulate the dysbiotic-metabolic-immune associations observed in this comorbidity.

### 4.6 Limitations and future directions

Several limitations of this study warrant consideration. First, its cross-sectional design precludes causal inference; the associations reported herein should be interpreted as hypothesis-generating and require validation in longitudinal cohorts. Second, although the sample size was informed by *a priori* power analysis, it remains modest and derived from a single center, which limits statistical power for multi-omics integration and necessitates cautious interpretation of the diagnostic model’s performance pending external replication in larger, multicenter populations. Third, the correlation analyzes presented are univariate and do not formally adjust for potential confounders such as age, sex, or detailed dietary parameters beyond baseline matching; while groups were well-matched on these characteristics (**Table 1**), residual confounding cannot be entirely excluded. Fourth, dietary assessment was limited to a baseline FFQ and a single 24-hour recall, which may not fully capture day-to-day variability in nutrient intake or long-term dietary patterns. Although the ARFC and HC groups were well-matched on key nutritional parameters—including total energy, total protein, animal/plant protein ratio, and dietary fiber intake (**Table 1**)—residual confounding by unmeasured dietary factors cannot be excluded. This is particularly relevant given that dietary protein sources have been shown to differentially modulate gut microbiome composition and metabolic output in children. A recent prospective cohort study of 1,826 Chinese children aged 6–8 years demonstrated that vegetable protein intake was positively associated with the abundance of SCFA-producing taxa (e.g., *Butyricicoccus, Dorea*), whereas higher animal protein consumption was associated with distinct microbial signatures (Xu et al., 2024). Furthermore, our recent work has proposed a GM-guided framework for stratified nutritional interventions in pediatric FC with comorbidities, underscoring the importance of diet-microbe interactions in this population (Huang et al., 2026). Future studies should incorporate more comprehensive dietary phenotyping—including repeated 24-hour recalls or food diaries—and formally adjust for dietary covariates in multivariate models to isolate the specific contributions of disease-associated dysbiosis from habitual dietary patterns Fifth, the metabolomic analysis was intentionally restricted to amino acids and their derivatives, omitting other critical classes of gut microbiota-derived metabolites—most notably SCFAs and bile acids—that are centrally involved in immune regulation and intestinal homeostasis (Arpaia et al., 2013; Parada Venegas et al., 2019; Yang et al., 2022; Yang et al., 2025). The immunomodulatory role of dietary fiber, the primary substrate for SCFA production, is further supported by an EAACI position paper (Venter et al., 2022). A recent comprehensive review highlights that SCFAs, bile acids, and amino acid metabolites together constitute a core network of microbial signals modulating allergic disease pathogenesis (Kim and Baker, 2026). Consequently, our metabolic characterization, while informative, is necessarily incomplete. Sixth, the use of rectal swabs rather than spontaneously voided stool samples introduces additional considerations; although this approach offered practical advantages for pediatric collection and has been validated in pediatric populations (Zhang et al., 2018; Fair et al., 2019; Reyman et al., 2019; K K et al., 2024; Perry et al., 2024), swabs sample mucosal-associated and luminal-interface communities rather than bulk luminal contents and may underestimate certain taxa (Fair et al., 2019) or fail to reliably capture specific metabolites (K K et al., 2024).

To address these limitations, future investigations should employ larger, multicenter prospective cohorts with repeated sampling and comprehensive dietary phenotyping to validate the identified associations and assess their temporal stability. Mechanistic studies utilizing gnotobiotic animal models with fecal microbiota transplantation from ARFC donors, coupled with untargeted metabolomics and metabolic flux analysis, are needed to establish causality and delineate molecular mechanisms. Furthermore, interventional studies exploring rationally designed probiotic or synbiotic formulations, as well as dietary modulation strategies targeting the dysbiotic-metabolic-immune axis delineated herein, represent promising translational avenues.

## 5 Conclusion

In conclusion, this integrated metagenomic and metabolomic analysis provides the first comprehensive characterization of the gut ecosystem in children with comorbid allergic rhinitis and functional constipation. We identified a convergent “gut microbiota-metabolism-immunity” axis underlying ARFC pathogenesis. The condition is marked by a synergistic dysbiosis—featuring an expansion of mucolytic Bacteroidota and a contraction of SCFA-producing Bacillota—coupled with a profound disruption of fecal amino acid homeostasis. Key immunomodulatory metabolites, such as glutamine and GABA, were depleted, and their related nutrient-sensing (mTOR, FoxO) and neuro-immune pathways were significantly enriched. Crucially, robust correlation networks directly linked this dysbiotic-metabolic landscape to clinical allergic sensitization (e.g., IgE levels). The exceptional discriminatory power of a combined microbial and metabolic model underscores its translational potential. Collectively, our findings advance the “gut-lung-enteric” axis concept, offering a novel multi-omics framework that not only elucidates the pathophysiology of ARFC but also pinpoints candidate targets for future microbiome-based diagnostics and interventions.

## Data Availability

The datasets presented in this study can be found in online repositories. The names of the repository/repositories and accession number(s) can be found in the article/**Supplementary Material**.
