# Supplementary figures and images for: Integrative metagenomic and metabolomic analysis reveals a gut microbiota-metabolite-immune axis in pediatric allergic rhinitis with functional constipation
Source: Front Cell Infect Microbiol. 2026 May 26;16:1779298. doi: 10.3389/fcimb.2026.1779298 (PMC13247438; doi:10.3389/fcimb.2026.1779298)

# Wilcoxon Rank sum test forshannonindex

p= 0.267

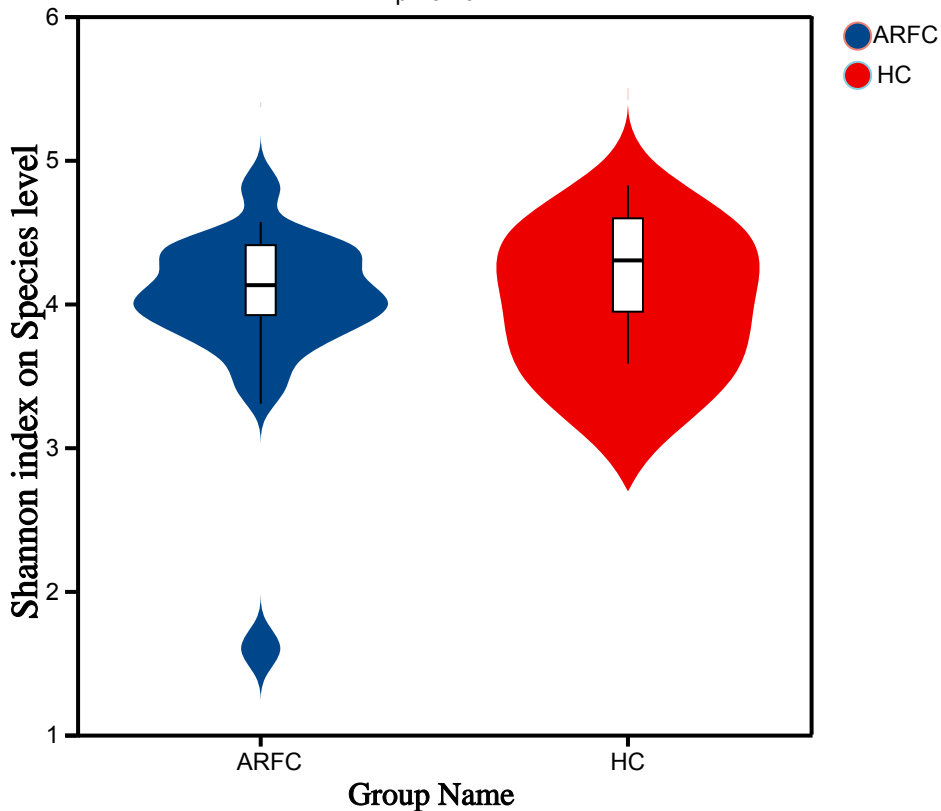

Supplement: Supplementary file 1 [file DataSheet1.pdf]
