# Supplementary material for: Integrative metagenomic and metabolomic analysis reveals a gut microbiota-metabolite-immune axis in pediatric allergic rhinitis with functional constipation
Source: Front Cell Infect Microbiol. 2026 May 26;16:1779298. doi: 10.3389/fcimb.2026.1779298 (PMC13247438; doi:10.3389/fcimb.2026.1779298)
Supplement: Supplementary file 2 [file Table1.docx]

| Num | First Category | Second Category | Pathway Desciption | Pathway_ID | Database | Enrichment ratio | DA Score | Ratio_in_study | Ratio_in_pop | P_value | P_adjust |
| --- | --- | --- | --- | --- | --- | --- | --- | --- | --- | --- | --- |
| 1 | Environmental Information Processing | Signal transduction | mTOR signaling pathway | map04150 | KEGG PATHWAY | 0.25 | 0.5 | 1/33 | 4/6496 | 0.0202 | 0.0474 |
| 1 | Environmental Information Processing | Signal transduction | FoxO signaling pathway | map04068 | KEGG PATHWAY | 0.2 | 1 | 1/33 | 5/6496 | 0.0252 | 0.0543 |
| 1 | Human Diseases | Infectious disease: bacterial | Epithelial cell signaling in Helicobacter pylori infection | map05120 | KEGG PATHWAY | 0.2 | -1 | 1/33 | 5/6496 | 0.0252 | 0.0543 |
| 1 | Human Diseases | Infectious disease: parasitic | Chagas disease | map05142 | KEGG PATHWAY | 0.1666 | 1 | 1/33 | 6/6496 | 0.0301 | 0.0614 |
| 1 | Human Diseases | Neurodegenerative disease | Huntington disease | map05016 | KEGG PATHWAY | 0.1666 | 1 | 1/33 | 6/6496 | 0.0301 | 0.0614 |
| 1 | Human Diseases | Neurodegenerative disease | Spinocerebellar ataxia | map05017 | KEGG PATHWAY | 0.1428 | 1 | 1/33 | 7/6496 | 0.0350 | 0.0676 |
| 1 | Human Diseases | Substance dependence | Cocaine addiction | map05030 | KEGG PATHWAY | 0.1428 | 0.5 | 1/33 | 7/6496 | 0.0350 | 0.0676 |
| 1 | Organismal Systems | Nervous system | Long-term potentiation | map04720 | KEGG PATHWAY | 0.1428 | 1 | 1/33 | 7/6496 | 0.0350 | 0.0676 |
| 1 | Human Diseases | Substance dependence | Morphine addiction | map05032 | KEGG PATHWAY | 0.125 | 0.5 | 1/33 | 8/6496 | 0.0400 | 0.0731 |
| 1 | Organismal Systems | Endocrine system | Estrogen signaling pathway | map04915 | KEGG PATHWAY | 0.125 | 0.5 | 1/33 | 8/6496 | 0.0400 | 0.0731 |
| 1 | Human Diseases | Infectious disease: parasitic | African trypanosomiasis | map05143 | KEGG PATHWAY | 0.125 | -0.5 | 1/33 | 8/6496 | 0.0400 | 0.0731 |
| 1 | Organismal Systems | Endocrine system | GnRH secretion | map04929 | KEGG PATHWAY | 0.1111 | 0.5 | 1/33 | 9/6496 | 0.0448 | 0.0769 |
| 1 | Organismal Systems | Nervous system | Long-term depression | map04730 | KEGG PATHWAY | 0.1111 | 1 | 1/33 | 9/6496 | 0.0448 | 0.0769 |
| 1 | Human Diseases | Substance dependence | Amphetamine addiction | map05031 | KEGG PATHWAY | 0.1111 | 0.5 | 1/33 | 9/6496 | 0.0448 | 0.0769 |
| 1 | Organismal Systems | Environmental adaptation | Circadian entrainment | map04713 | KEGG PATHWAY | 0.1111 | 1 | 1/33 | 9/6496 | 0.0448 | 0.0769 |
| 1 | Human Diseases | Substance dependence | Alcoholism | map05034 | KEGG PATHWAY | 0.1 | 0.5 | 1/33 | 10/6496 | 0.0497 | 0.0789 |
| 1 | Metabolism | Biosynthesis of other secondary metabolites | Clavulanic acid biosynthesis | map00331 | KEGG PATHWAY | 0.1 | 1 | 1/33 | 10/6496 | 0.0497 | 0.0789 |
| 1 | Environmental Information Processing | Signal transduction | Phospholipase D signaling pathway | map04072 | KEGG PATHWAY | 0.0909 | 1 | 1/33 | 11/6496 | 0.0545 | 0.0841 |
| 1 | Cellular Processes | Cellular community - eukaryotes | Gap junction | map04540 | KEGG PATHWAY | 0.0909 | 0.5 | 1/33 | 11/6496 | 0.0545 | 0.0841 |
| 1 | Human Diseases | Infectious disease: parasitic | Amoebiasis | map05146 | KEGG PATHWAY | 0.07692 | 1 | 1/33 | 13/6496 | 0.0641 | 0.0962 |
| 1 | Human Diseases | Infectious disease: bacterial | Shigellosis | map05131 | KEGG PATHWAY | 0.07142 | -0.5 | 1/33 | 14/6496 | 0.0689 | 0.1005 |
| 1 | Environmental Information Processing | Signal transduction | Sphingolipid signaling pathway | map04071 | KEGG PATHWAY | 0.06666 | -0.5 | 1/33 | 15/6496 | 0.0736 | 0.1060 |
| 1 | Human Diseases | Drug resistance: antineoplastic | Antifolate resistance | map01523 | KEGG PATHWAY | 0.05882 | -0.5 | 1/33 | 17/6496 | 0.0830 | 0.1180 |
| 1 | Metabolism | Biosynthesis of other secondary metabolites | Prodigiosin biosynthesis | map00333 | KEGG PATHWAY | 0.05555 | -1 | 1/33 | 18/6496 | 0.0877 | 0.1230 |
| 1 | Organismal Systems | Endocrine system | Thyroid hormone synthesis | map04918 | KEGG PATHWAY | 0.04761 | -1 | 1/33 | 21/6496 | 0.1016 | 0.1389 |
| 1 | Metabolism | Xenobiotics biodegradation and metabolism | Atrazine degradation | map00791 | KEGG PATHWAY | 0.04347 | -1 | 1/33 | 23/6496 | 0.1107 | 0.1495 |
| 1 | Metabolism | Energy metabolism | Carbon fixation in photosynthetic organisms | map00710 | KEGG PATHWAY | 0.04347 | -0.3333 | 1/33 | 23/6496 | 0.1107 | 0.1495 |
| 1 | Environmental Information Processing | Signal transduction | cAMP signaling pathway | map04024 | KEGG PATHWAY | 0.04 | 0.25 | 1/33 | 25/6496 | 0.1198 | 0.1577 |
| 1 | Metabolism | Xenobiotics biodegradation and metabolism | Caprolactam degradation | map00930 | KEGG PATHWAY | 0.03846 | 1 | 1/33 | 26/6496 | 0.1242 | 0.1617 |
| 1 | Metabolism | Glycan biosynthesis and metabolism | Peptidoglycan biosynthesis | map00550 | KEGG PATHWAY | 0.03703 | -1 | 1/33 | 27/6496 | 0.1287 | 0.1655 |
| 1 | Metabolism | Metabolism of other amino acids | Selenocompound metabolism | map00450 | KEGG PATHWAY | 0.03703 | -1 | 1/33 | 27/6496 | 0.1287 | 0.1655 |
| 1 | Metabolism | Metabolism of cofactors and vitamins | Vitamin B6 metabolism | map00750 | KEGG PATHWAY | 0.03448 | -1 | 1/33 | 29/6496 | 0.1376 | 0.1728 |
| 1 | Metabolism | Metabolism of cofactors and vitamins | Biotin metabolism | map00780 | KEGG PATHWAY | 0.03448 | -0.5 | 1/33 | 29/6496 | 0.1376 | 0.1728 |
| 1 | Metabolism | Metabolism of cofactors and vitamins | Thiamine metabolism | map00730 | KEGG PATHWAY | 0.03225 | -0.3333 | 1/33 | 31/6496 | 0.1464 | 0.1796 |
| 1 | Metabolism | Biosynthesis of other secondary metabolites | Novobiocin biosynthesis | map00401 | KEGG PATHWAY | 0.03125 | -0.5 | 1/33 | 32/6496 | 0.1507 | 0.1829 |
| 1 | Metabolism | Chemical structure transformation maps | Biosynthesis of alkaloids derived from histidine and purine | map01065 | KEGG PATHWAY | 0.02777 | -0.3333 | 1/33 | 36/6496 | 0.1679 | 0.1929 |
| 1 | Metabolism | Carbohydrate metabolism | C5-Branched dibasic acid metabolism | map00660 | KEGG PATHWAY | 0.02857 | 1 | 1/33 | 35/6496 | 0.1637 | 0.1964 |
| 1 | Metabolism | Amino acid metabolism | Phenylalanine, tyrosine and tryptophan biosynthesis | map00400 | KEGG PATHWAY | 0.02857 | -0.3333 | 1/33 | 35/6496 | 0.1637 | 0.1964 |
| 1 | Metabolism | Lipid metabolism | Sphingolipid metabolism | map00600 | KEGG PATHWAY | 0.02857 | -0.5 | 1/33 | 35/6496 | 0.1637 | 0.1964 |
| 1 | Metabolism | Lipid metabolism | Primary bile acid biosynthesis | map00120 | KEGG PATHWAY | 0.02127 | 0.3333 | 1/33 | 47/6496 | 0.2135 | 0.2378 |
| 1 | Metabolism | Amino acid metabolism | Phenylalanine metabolism | map00360 | KEGG PATHWAY | 0.0204 | -0.3333 | 1/33 | 49/6496 | 0.2216 | 0.2442 |
| 1 | Cellular Processes | Cellular community - prokaryotes | Quorum sensing | map02024 | KEGG PATHWAY | 0.01818 | 0.5 | 1/33 | 55/6496 | 0.2452 | 0.2675 |
| 1 | Metabolism | Lipid metabolism | Glycerophospholipid metabolism | map00564 | KEGG PATHWAY | 0.01785 | -0.5 | 1/33 | 56/6496 | 0.2491 | 0.2690 |
| 1 | Human Diseases | Cancer: overview | Chemical carcinogenesis - reactive oxygen species | map05208 | KEGG PATHWAY | 0.01754 | -1 | 1/33 | 57/6496 | 0.2529 | 0.2704 |
| 1 | Metabolism | Biosynthesis of other secondary metabolites | Phenylpropanoid biosynthesis | map00940 | KEGG PATHWAY | 0.01724 | -0.5 | 1/33 | 58/6496 | 0.2567 | 0.2718 |
| 1 | Metabolism | Global and overview maps | Nucleotide metabolism | map01232 | KEGG PATHWAY | 0.01724 | -1 | 1/33 | 58/6496 | 0.2567 | 0.2718 |
| 1 | Metabolism | Biosynthesis of other secondary metabolites | Biosynthesis of various alkaloids | map00996 | KEGG PATHWAY | 0.01315 | -0.5 | 1/33 | 76/6496 | 0.3225 | 0.3349 |
| 1 | Metabolism | Amino acid metabolism | Tyrosine metabolism | map00350 | KEGG PATHWAY | 0.01282 | -0.3333 | 1/33 | 78/6496 | 0.3294 | 0.3388 |
| 1 | Metabolism | Biosynthesis of other secondary metabolites | Neomycin, kanamycin and gentamicin biosynthesis | map00524 | KEGG PATHWAY | 0.01234 | 1 | 1/33 | 81/6496 | 0.3397 | 0.3461 |
| 1 | Metabolism | Energy metabolism | Methane metabolism | map00680 | KEGG PATHWAY | 0.01136 | -0.2 | 1/33 | 88/6496 | 0.3631 | 0.3665 |
| 1 | Metabolism | Chemical structure transformation maps | Biosynthesis of phenylpropanoids | map01061 | KEGG PATHWAY | 0.009345 | -0.3333 | 1/33 | 107/6496 | 0.4227 | 0.4227 |
| 2 | Human Diseases | Substance dependence | Nicotine addiction | map05033 | KEGG PATHWAY | 0.2857 | 0.6667 | 2/33 | 7/6496 | 0.0005 | 0.0027 |
| 2 | Organismal Systems | Nervous system | Glutamatergic synapse | map04724 | KEGG PATHWAY | 0.25 | 0 | 2/33 | 8/6496 | 0.0007 | 0.0030 |
| 2 | Genetic Information Processing | Folding, sorting and degradation | Sulfur relay system | map04122 | KEGG PATHWAY | 0.1818 | -1 | 2/33 | 11/6496 | 0.0013 | 0.0052 |
| 2 | Organismal Systems | Nervous system | Synaptic vesicle cycle | map04721 | KEGG PATHWAY | 0.1666 | 0.4 | 2/33 | 12/6496 | 0.0016 | 0.0060 |
| 2 | Human Diseases | Neurodegenerative disease | Amyotrophic lateral sclerosis | map05014 | KEGG PATHWAY | 0.1428 | 1 | 2/33 | 14/6496 | 0.0022 | 0.0079 |
| 2 | Organismal Systems | Excretory system | Proximal tubule bicarbonate reclamation | map04964 | KEGG PATHWAY | 0.1176 | 0 | 2/33 | 17/6496 | 0.0032 | 0.0106 |
| 2 | Metabolism | Biosynthesis of other secondary metabolites | Penicillin and cephalosporin biosynthesis | map00311 | KEGG PATHWAY | 0.1111 | -0.6667 | 2/33 | 18/6496 | 0.0036 | 0.0116 |
| 2 | Organismal Systems | Nervous system | Retrograde endocannabinoid signaling | map04723 | KEGG PATHWAY | 0.1052 | 0.6667 | 2/33 | 19/6496 | 0.0041 | 0.0122 |
| 2 | Metabolism | Energy metabolism | Nitrogen metabolism | map00910 | KEGG PATHWAY | 0.1052 | 0 | 2/33 | 19/6496 | 0.0041 | 0.0122 |
| 2 | Cellular Processes | Cell growth and death | Ferroptosis | map04216 | KEGG PATHWAY | 0.06896 | 0 | 2/33 | 29/6496 | 0.0093 | 0.0258 |
| 2 | Metabolism | Metabolism of cofactors and vitamins | Pantothenate and CoA biosynthesis | map00770 | KEGG PATHWAY | 0.06666 | -0.4 | 2/33 | 30/6496 | 0.0100 | 0.0269 |
| 2 | Metabolism | Metabolism of other amino acids | beta-Alanine metabolism | map00410 | KEGG PATHWAY | 0.0625 | 0 | 2/33 | 32/6496 | 0.0113 | 0.0297 |
| 2 | Organismal Systems | Sensory system | Taste transduction | map04742 | KEGG PATHWAY | 0.0625 | 0.5 | 2/33 | 32/6496 | 0.0113 | 0.0297 |
| 2 | Human Diseases | Neurodegenerative disease | Pathways of neurodegeneration - multiple diseases | map05022 | KEGG PATHWAY | 0.0625 | 1 | 2/33 | 32/6496 | 0.0113 | 0.0297 |
| 2 | Metabolism | Metabolism of other amino acids | Glutathione metabolism | map00480 | KEGG PATHWAY | 0.05263 | 0 | 2/33 | 38/6496 | 0.0157 | 0.0385 |
| 2 | Metabolism | Biosynthesis of other secondary metabolites | Staurosporine biosynthesis | map00404 | KEGG PATHWAY | 0.04166 | -0.6667 | 2/33 | 48/6496 | 0.0244 | 0.0538 |
| 2 | Metabolism | Carbohydrate metabolism | Butanoate metabolism | map00650 | KEGG PATHWAY | 0.04255 | 0.5 | 2/33 | 47/6496 | 0.0235 | 0.0539 |
| 2 | Metabolism | Amino acid metabolism | Histidine metabolism | map00340 | KEGG PATHWAY | 0.04255 | 0 | 2/33 | 47/6496 | 0.0235 | 0.0539 |
| 2 | Environmental Information Processing | Signal transduction | Two-component system | map02020 | KEGG PATHWAY | 0.03773 | 0 | 2/33 | 53/6496 | 0.0294 | 0.0610 |
| 2 | Metabolism | Metabolism of cofactors and vitamins | Nicotinate and nicotinamide metabolism | map00760 | KEGG PATHWAY | 0.03636 | 0 | 2/33 | 55/6496 | 0.0314 | 0.0617 |
| 2 | Metabolism | Nucleotide metabolism | Pyrimidine metabolism | map00240 | KEGG PATHWAY | 0.03125 | -0.6667 | 2/33 | 64/6496 | 0.0415 | 0.0723 |
| 2 | Metabolism | Chemical structure transformation maps | Biosynthesis of plant hormones | map01070 | KEGG PATHWAY | 0.02898 | -0.3333 | 2/33 | 69/6496 | 0.0475 | 0.0766 |
| 2 | Metabolism | Amino acid metabolism | Tryptophan metabolism | map00380 | KEGG PATHWAY | 0.02409 | -0.4 | 2/33 | 83/6496 | 0.0660 | 0.0976 |
| 2 | Metabolism | Nucleotide metabolism | Purine metabolism | map00230 | KEGG PATHWAY | 0.0198 | -0.6667 | 2/33 | 101/6496 | 0.0926 | 0.1282 |
| 2 | Metabolism | Chemical structure transformation maps | Biosynthesis of alkaloids derived from shikimate pathway | map01063 | KEGG PATHWAY | 0.01388 | -0.2857 | 2/33 | 144/6496 | 0.1655 | 0.1922 |
| 2 | Metabolism | Metabolism of cofactors and vitamins | Porphyrin metabolism | map00860 | KEGG PATHWAY | 0.01342 | 0 | 2/33 | 149/6496 | 0.1745 | 0.1984 |
| 2 | Metabolism | Biosynthesis of other secondary metabolites | Biosynthesis of various plant secondary metabolites | map00999 | KEGG PATHWAY | 0.01342 | -0.3333 | 2/33 | 149/6496 | 0.1745 | 0.1984 |
| 3 | Organismal Systems | Nervous system | GABAergic synapse | map04727 | KEGG PATHWAY | 0.3333 | 0.2 | 3/33 | 9/6496 | 9.83E-06 | 7.08E-05 |
| 3 | Metabolism | Amino acid metabolism | Valine, leucine and isoleucine biosynthesis | map00290 | KEGG PATHWAY | 0.1304 | -0.75 | 3/33 | 23/6496 | 0.0002 | 0.0012 |
| 3 | Human Diseases | Drug resistance: antimicrobial | Vancomycin resistance | map01502 | KEGG PATHWAY | 0.125 | -0.75 | 3/33 | 24/6496 | 0.0002 | 0.0013 |
| 3 | Metabolism | Biosynthesis of other secondary metabolites | Carbapenem biosynthesis | map00332 | KEGG PATHWAY | 0.09375 | -0.3333 | 3/33 | 32/6496 | 0.0005 | 0.0026 |
| 3 | Metabolism | Energy metabolism | Sulfur metabolism | map00920 | KEGG PATHWAY | 0.0909 | -0.1429 | 3/33 | 33/6496 | 0.0006 | 0.0026 |
| 3 | Metabolism | Biosynthesis of other secondary metabolites | Monobactam biosynthesis | map00261 | KEGG PATHWAY | 0.07692 | -0.1667 | 3/33 | 39/6496 | 0.0010 | 0.0040 |
| 3 | Metabolism | Amino acid metabolism | Valine, leucine and isoleucine degradation | map00280 | KEGG PATHWAY | 0.07142 | -0.75 | 3/33 | 42/6496 | 0.0012 | 0.0048 |
| 3 | Environmental Information Processing | Signaling molecules and interaction | Neuroactive ligand-receptor interaction | map04080 | KEGG PATHWAY | 0.0566 | 0.2727 | 3/33 | 53/6496 | 0.0024 | 0.0082 |
| 3 | Metabolism | Amino acid metabolism | Lysine degradation | map00310 | KEGG PATHWAY | 0.05357 | -0.1111 | 3/33 | 56/6496 | 0.0028 | 0.0093 |
| 3 | Metabolism | Carbohydrate metabolism | Glyoxylate and dicarboxylate metabolism | map00630 | KEGG PATHWAY | 0.04687 | -0.2 | 3/33 | 64/6496 | 0.0040 | 0.0124 |
| 3 | Metabolism | Biosynthesis of other secondary metabolites | Tropane, piperidine and pyridine alkaloid biosynthesis | map00960 | KEGG PATHWAY | 0.04166 | -0.2 | 3/33 | 72/6496 | 0.0056 | 0.0160 |
| 3 | Metabolism | Biosynthesis of other secondary metabolites | Biosynthesis of various antibiotics | map00998 | KEGG PATHWAY | 0.02752 | 0.125 | 3/33 | 109/6496 | 0.0174 | 0.0418 |
| 4 | Metabolism | Amino acid metabolism | Arginine biosynthesis | map00220 | KEGG PATHWAY | 0.1739 | 0 | 4/33 | 23/6496 | 4.57E-06 | 3.79E-05 |
| 4 | Metabolism | Metabolism of other amino acids | Taurine and hypotaurine metabolism | map00430 | KEGG PATHWAY | 0.1666 | 0 | 4/33 | 24/6496 | 5.46E-06 | 4.21E-05 |
| 4 | Metabolism | Biosynthesis of other secondary metabolites | Biosynthesis of various other secondary metabolites | map00997 | KEGG PATHWAY | 0.05882 | -0.1818 | 4/33 | 68/6496 | 0.0004 | 0.0019 |
| 4 | Metabolism | Biosynthesis of other secondary metabolites | Glucosinolate biosynthesis | map00966 | KEGG PATHWAY | 0.05194 | -0.5714 | 4/33 | 77/6496 | 0.0006 | 0.0027 |
| 5 | Metabolism | Amino acid metabolism | Alanine, aspartate and glutamate metabolism | map00250 | KEGG PATHWAY | 0.1785 | -0.0909 | 5/33 | 28/6496 | 2.23E-07 | 2.41E-06 |
| 5 | Metabolism | Metabolism of other amino acids | Cyanoamino acid metabolism | map00460 | KEGG PATHWAY | 0.1111 | -0.4167 | 5/33 | 45/6496 | 2.61E-06 | 2.56E-05 |
| 5 | Metabolism | Amino acid metabolism | Glycine, serine and threonine metabolism | map00260 | KEGG PATHWAY | 0.1041 | -0.3846 | 5/33 | 48/6496 | 3.62E-06 | 3.26E-05 |
| 5 | Metabolism | Chemical structure transformation maps | Biosynthesis of alkaloids derived from ornithine, lysine and nicotinic acid | map01064 | KEGG PATHWAY | 0.07352 | 0.09091 | 5/33 | 68/6496 | 2.05E-05 | 0.0001 |
| 7 | Metabolism | Amino acid metabolism | Cysteine and methionine metabolism | map00270 | KEGG PATHWAY | 0.1044 | -0.4667 | 7/33 | 67/6496 | 3.12E-08 | 3.74E-07 |
| 8 | Metabolism | Amino acid metabolism | Arginine and proline metabolism | map00330 | KEGG PATHWAY | 0.1159 | 0 | 8/33 | 69/6496 | 1.20E-09 | 1.62E-08 |
| 9 | Organismal Systems | Digestive system | Mineral absorption | map04978 | KEGG PATHWAY | 0.3103 | -0.6429 | 9/33 | 29/6496 | 6.40E-15 | 1.15E-13 |
| 9 | Metabolism | Global and overview maps | Biosynthesis of cofactors | map01240 | KEGG PATHWAY | 0.02735 | -0.4667 | 9/33 | 329/6496 | 2.57E-05 | 0.0002 |
| 12 | Human Diseases | Cancer: overview | Central carbon metabolism in cancer | map05230 | KEGG PATHWAY | 0.3243 | -0.381 | 12/33 | 37/6496 | 5.23E-20 | 1.88E-18 |
| 13 | Organismal Systems | Digestive system | Protein digestion and absorption | map04974 | KEGG PATHWAY | 0.2765 | -0.375 | 13/33 | 47/6496 | 1.26E-20 | 1.36E-18 |
| 13 | Genetic Information Processing | Translation | Aminoacyl-tRNA biosynthesis | map00970 | KEGG PATHWAY | 0.25 | -0.3913 | 13/33 | 52/6496 | 5.59E-20 | 1.51E-18 |
| 13 | Metabolism | Chemical structure transformation maps | Biosynthesis of plant secondary metabolites | map01060 | KEGG PATHWAY | 0.09154 | -0.2692 | 13/33 | 142/6496 | 5.90E-14 | 9.10E-13 |
| 14 | Metabolism | Metabolism of other amino acids | D-Amino acid metabolism | map00470 | KEGG PATHWAY | 0.2028 | -0.4211 | 14/33 | 69/6496 | 4.04E-20 | 2.18E-18 |
| 14 | Environmental Information Processing | Membrane transport | ABC transporters | map02010 | KEGG PATHWAY | 0.1014 | -0.2727 | 14/33 | 138/6496 | 1.14E-15 | 2.46E-14 |
