# Supplementary material for: Integrative metagenomic and metabolomic analysis reveals a gut microbiota-metabolite-immune axis in pediatric allergic rhinitis with functional constipation
Source: Front Cell Infect Microbiol. 2026 May 26;16:1779298. doi: 10.3389/fcimb.2026.1779298 (PMC13247438; doi:10.3389/fcimb.2026.1779298)
Supplement: Supplementary file 3 [file Table2.docx]

| Name | total ige | Dust mites | milk | Eggs | shrimp | crabs | dog hairs | cat hairs | beef |
| --- | --- | --- | --- | --- | --- | --- | --- | --- | --- |
| s__Bacteroides_fragilis | 0.2185 | 0.0798 | 0.1312 | -0.1401 | 0.2376 | 0.2335 | 0.0169 | -0.1708 | 0.3135 |
| s__Bacteroides_sp. | 0.3175 | 0.2134 | 0.1928 | -0.0817 | 0.2823 | 0.2802 | 0.0777 | -0.2197 | 0.3478 |
| s__Streptococcus_sp. | -0.7156 | -0.4592 | -0.4341 | -0.2568 | -0.2797 | -0.2802 | -0.0818 | 0.0081 | -0.3392 |
| s__Lachnospira_eligens | 0.1355 | -0.0044 | -0.2337 | 0.1868 | -0.0661 | -0.0584 | -0.2000 | 0.1058 | -0.1940 |
| s__Alistipes_sp. | 0.0076 | -0.0157 | -0.0560 | 0.1518 | 0.0078 | 0.0117 | 0.0452 | 0.1546 | 0.0274 |
| s__Phocaeicola_vulgatus | 0.5180 | 0.2712 | 0.1834 | 0.0700 | 0.2830 | 0.2802 | -0.0131 | -0.1546 | 0.2352 |
| s__Oscillibacter_sp. | 0.0343 | 0.0803 | 0.0543 | 0.2802 | 0.0194 | 0.0117 | -0.0903 | 0.2685 | 0.0454 |
| s__Bifidobacterium_longum | -0.3902 | -0.3495 | -0.1650 | -0.2568 | -0.1650 | -0.1634 | -0.1546 | -0.1220 | -0.0565 |
| s__Bacteriophage_sp. | -0.0971 | 0.0005 | -0.0328 | 0.2101 | -0.0603 | -0.0584 | -0.0319 | 0.1220 | 0.0553 |
| s__Gemmiger_sp. | -0.0335 | 0.1562 | -0.1079 | 0.3035 | -0.0982 | -0.0934 | -0.0473 | 0.0407 | -0.0994 |
| s__Escherichia_coli | 0.2100 | 0.2492 | -0.0359 | 0.1051 | 0.1005 | 0.1051 | 0.0482 | 0.0732 | -0.0741 |
| s__Proteus_sp. | 0.2719 | 0.3015 | -0.0492 | 0.1753 | 0.0432 | 0.0468 | -0.0277 | 0.0570 | -0.1235 |
| s__Phocaeicola_sp. | 0.5551 | 0.2932 | 0.2427 | 0.0817 | 0.2596 | 0.2568 | -0.0105 | -0.0732 | 0.2386 |
| s__Faecalibacterium_sp. | 0.1907 | 0.1787 | -0.0590 | 0.2218 | -0.1617 | -0.1634 | -0.2359 | 0.1546 | -0.1709 |
| s__Faecalibacterium_prausnitzii | 0.1578 | 0.0666 | 0.0800 | 0.1518 | 0.0263 | 0.0233 | -0.1648 | 0.0081 | 0.0587 |
| s__Eubacterium_sp. | 0.0030 | 0.0915 | -0.1358 | 0.1751 | -0.0655 | -0.0584 | 0.0602 | 0.2197 | -0.3028 |
| s__Fusicatenibacter_saccharivorans | -0.1633 | 0.0078 | -0.2578 | 0.0350 | -0.1342 | -0.1284 | -0.1051 | 0.0895 | -0.0728 |
| s__Bifidobacterium_pseudocatenulatum | -0.3948 | -0.2923 | -0.2656 | -0.2101 | 0.1037 | 0.1051 | -0.0150 | -0.0081 | 0.0283 |
| s__Akkermansia_sp. | 0.0084 | 0.0211 | 0.2935 | 0.0000 | 0.2810 | 0.2802 | 0.1869 | 0.1546 | 0.4091 |
| s__Roseburia_sp. | -0.0234 | 0.0676 | -0.3011 | 0.3035 | -0.0535 | -0.0467 | -0.0856 | 0.0895 | -0.1820 |
| s__Gallintestinimicrobium_sp. | -0.3654 | -0.0436 | -0.1047 | 0.2568 | -0.3270 | -0.3269 | -0.0554 | 0.1220 | -0.1276 |
| s__Bacteroides_uniformis | 0.1820 | 0.0485 | 0.0950 | -0.2218 | 0.3164 | 0.3152 | 0.0942 | -0.1546 | 0.3204 |
| s__Mediterraneibacter_sp. | -0.2359 | -0.0494 | -0.1223 | 0.0584 | 0.1809 | 0.1751 | 0.0983 | -0.2197 | 0.0994 |
| s__Ruminococcus_sp. | -0.1945 | -0.0088 | -0.1529 | 0.2335 | 0.0259 | 0.0350 | -0.0732 | 0.1058 | -0.0964 |
| s__Flavonifractor_plautii | -0.1793 | -0.1581 | 0.0365 | -0.1167 | 0.3258 | 0.3269 | -0.1398 | -0.2197 | 0.2454 |
| s__Agathobacter_rectalis | -0.1888 | -0.1322 | -0.5088 | 0.0584 | -0.1977 | -0.1984 | -0.2209 | 0.2359 | -0.4318 |
| s__Roseburia_inulinivorans | -0.1975 | -0.1527 | -0.4423 | 0.1518 | -0.0548 | -0.0467 | -0.1820 | 0.0732 | -0.3080 |
| s__Mediterraneibacter_gnavus | -0.2217 | -0.1146 | -0.0890 | -0.3269 | 0.2155 | 0.2101 | 0.1049 | -0.1383 | 0.1636 |
| s__Ruthenibacterium_lactatiformans | -0.4261 | -0.0259 | -0.0355 | -0.0817 | -0.1018 | -0.1051 | 0.2321 | 0.0732 | 0.0356 |
| s__Anaerostipes_hadrus | -0.4544 | -0.1953 | -0.3562 | -0.0584 | -0.1523 | -0.1518 | -0.1627 | -0.0569 | -0.3461 |
| s__Parabacteroides_sp. | 0.2560 | 0.0788 | 0.1672 | -0.1051 | 0.3270 | 0.3269 | 0.1944 | 0.2034 | 0.3851 |
| s__Bacteroides_xylanisolvens | 0.1842 | 0.0206 | -0.0333 | -0.2101 | 0.2023 | 0.1984 | -0.1869 | -0.1708 | 0.1186 |
| s__Gallintestinimicrobium_propionicum | -0.2985 | -0.0313 | -0.2045 | 0.2568 | -0.2710 | -0.2685 | -0.0362 | 0.1220 | -0.1469 |
| s__Bifidobacterium_breve | -0.5047 | -0.3158 | -0.2213 | -0.3269 | -0.2230 | -0.2218 | 0.0030 | -0.1708 | -0.1002 |
| s__Blautia_wexlerae | -0.5676 | -0.2996 | -0.4731 | -0.0467 | -0.1523 | -0.1518 | -0.2012 | -0.1546 | -0.3748 |
| s__Segatella_copri | 0.2683 | 0.2409 | 0.2743 | 0.2918 | 0.2237 | 0.2218 | 0.0706 | 0.2522 | 0.1975 |
| s__Gemmiger_formicilis | -0.1491 | 0.1674 | -0.1598 | 0.1868 | -0.1115 | -0.1051 | 0.0148 | 0.0569 | -0.0480 |
| s__Akkermansia_muciniphila | 0.0433 | -0.0255 | 0.2291 | -0.0817 | 0.3157 | 0.3152 | 0.1374 | 0.1708 | 0.3247 |
| s__Flavonifractor_sp. | -0.1657 | -0.1620 | -0.0435 | -0.1868 | 0.3377 | 0.3385 | -0.1381 | -0.2522 | 0.2390 |
| s__Bifidobacterium_sp. | -0.5006 | -0.4342 | -0.2970 | -0.2802 | -0.0603 | -0.0584 | -0.1404 | -0.1220 | -0.0801 |
| s__Caudoviricetes_sp. | -0.1162 | 0.0416 | -0.0062 | 0.2335 | 0.0810 | 0.0817 | 0.0011 | 0.2359 | 0.0231 |
| s__Anaerostipes_sp. | -0.5425 | -0.2746 | -0.3600 | -0.1518 | -0.2564 | -0.2568 | -0.1314 | -0.1383 | -0.3281 |
| s__Proteus_mirabilis | 0.1540 | 0.0754 | -0.2296 | 0.1634 | -0.2003 | -0.1984 | -0.2477 | 0.0407 | -0.4450 |
| s__Blautia_sp. | -0.5771 | -0.2888 | -0.4628 | -0.0700 | -0.2343 | -0.2335 | -0.1317 | -0.0732 | -0.4103 |
| s__Phocaeicola_dorei | 0.3790 | 0.1767 | 0.2621 | 0.1518 | 0.1190 | 0.1167 | -0.0028 | -0.1383 | 0.1623 |
| s__Bacteroides_thetaiotaomicron | 0.5447 | 0.4225 | 0.4875 | 0.0817 | 0.1776 | 0.1751 | 0.2353 | -0.1871 | 0.4622 |
| s__Bacteroides_ovatus | 0.1785 | 0.0078 | -0.0863 | -0.2101 | 0.2029 | 0.1984 | -0.1177 | -0.1546 | 0.1649 |
| s__Clostridium_sp. | -0.2669 | 0.0460 | -0.2934 | 0.0584 | -0.2810 | -0.2802 | -0.1107 | 0.2847 | -0.2202 |
| s__Phocaeicola_plebeius | 0.4030 | 0.1278 | 0.1786 | -0.1401 | 0.3251 | 0.3269 | 0.0032 | 0.2197 | 0.2373 |
| s__Agathobacter_sp. | -0.2062 | -0.0250 | -0.4369 | 0.1051 | -0.2136 | -0.2101 | -0.1484 | 0.2359 | -0.3607 |
