# Supplementary material for: Integrative metagenomic and metabolomic analysis reveals a gut microbiota-metabolite-immune axis in pediatric allergic rhinitis with functional constipation
Source: Front Cell Infect Microbiol. 2026 May 26;16:1779298. doi: 10.3389/fcimb.2026.1779298 (PMC13247438; doi:10.3389/fcimb.2026.1779298)
Supplement: Supplementary file 4 [file Table3.docx]

Supplementary Table S3. Clinical characteristics of the ARFC group.

| Characteristic | ARFC group (n=19) |
| --- | --- |
| Disease duration (months), median (IQR) | 14.5 (8.0–22.0) |
| TNSS, mean ± SD | 7.8 ± 2.1 |
| ARIA classification (intermittent/persistent), n | 7 / 12 |
| Defecation frequency (stools/week), mean ± SD | 2.8 ± 1.1 |
| Bristol Stool Form Scale (type 1–2), n (%) | 16 (84.2%) |
| Abdominal pain (≥1 episode/week), n (%) | 9 (47.4%) |
| Current AR medication use, n (%) | 0 (0%) |
| Current FC medication use, n (%) | 0 (0%) |
